# Non-linear association of atherogenic index of plasma with insulin resistance and type 2 diabetes: a cross-sectional study

**DOI:** 10.1186/s12933-023-01886-5

**Published:** 2023-06-29

**Authors:** Bei Yin, Zihong Wu, Yaqing Xia, Shunqiang Xiao, Lingling Chen, Yousheng Li

**Affiliations:** 1 Department of Traditional Chinese Medicine, Shenzhen People’s Hospital (The Second Clinical Medical College, Jinan University; The First Affiliated Hospital, Southern University of Science and Technology), Shenzhen, 518020 China; 2 Hospital of Chengdu University of Traditional Chinese Medicine, Chengdu, 610000 China; 3 Department of Endocrinology, Guangdong Provincial Hospital of Chinese Medicine (The Second Affiliated Hospital, Guangzhou University of Chinese Medicine), Guangzhou, 510000 China

**Keywords:** Atherogenic index of plasma, Insulin resistance, Type 2 diabetes, Cross-sectional study, NHANES

## Abstract

**Background:**

Although there is numerous evidence on the epidemiological risk factors for insulin resistance (IR)-related metabolic diseases, there is still insufficient evidence to explore the non-linear association of Atherogenic Index of Plasma (AIP) with IR. Therefore, we aimed to elucidate the non-linear relationship between AIP and IR and type 2 diabetes (T2D).

**Methods:**

This cross-sectional study was conducted in the National Health and Nutrition Survey (NHANES) from 2009 to 2018. A total of 9,245 participants were included in the study. The AIP was calculated as log10 (triglycerides/high-density lipoprotein cholesterol). The outcome variables included IR and T2D defined by the 2013 American Diabetes Association guidelines. The weighted multivariate linear regression, weighted multivariate logistic regression, subgroup analysis, generalized additive model, smooth fitting curve and two-part logistic regression were adopted to reveal the relationship of AIP with IR and T2D.

**Results:**

After adjustment for age, gender, race, education level, smoking status, alcohol consumption, vigorous/moderate physical activity, body mass index, waist circumference and hypertension, we found that AIP was positively associated with fasting blood glucose (β = 0.08, 95% CI: 0.06, 0.10), glycosylated hemoglobin (β = 0.04, 95% CI: 0.39, 0.58), fasting serum insulin (β = 4.26, 95% CI: 3.73, 4.79), and homeostasis model assessment of insulin resistance (β = 0.22, 95% CI: 0.18, 0.25). Further studies found that AIP was associated with increased risk of IR (OR = 1.29, 95% CI: 1.26–1.32) and T2D (OR = 1.18, 95% CI: 1.15–1.22). However, the positive association between AIP and IR or T2D was more significant in female than in male (IR: P for interaction = 0.0135; T2D: P for interaction = 0.0024). A non-linear and inverse L-shaped association was found between AIP and IR, while a J-shaped association was found between AIP and T2D. In patients with − 0.47 < AIP < 0.45, increased AIP was significantly associated with increased risk of IR and T2D.

**Conclusions:**

AIP showed an inverse L-shaped association with IR and a J-shaped association with T2D, indicating that AIP should be reduced to a certain level to prevent IR and T2D.

**Supplementary Information:**

The online version contains supplementary material available at 10.1186/s12933-023-01886-5.

## Introduction

Individuals with insulin resistance (IR), such as abdominal obesity, type 2 diabetes (T2D) and metabolic syndrome, usually have dyslipidemia, resulting in an increased risk of atherosclerotic cardiovascular disease (ASCVD). Typical dyslipidemia of these diseases are hypertriglyceridemia, elevated small-dense low density lipoprotein (sdLDL) particles, decreased numbers of high-density lipoprotein cholesterol (HDL-C) and high-density lipoprotein particles, elevated residual lipoprotein, and postprandial hyperlipidemia. These abnormalities are collectively referred to as atherogenic dyslipidemia complex (ADC) [1].

Among them, sdLDL is a subclass isolated from other low-density lipoprotein (LDL) by ultracentrifugation or gradient gel electrophoresis. There is ample evidence that sdLDL has a greater atherogenic potential than other LDL subclasses [2]. Circulating sdLDL is more susceptible to atherogenic modifications (catabolism, glycation, and oxidation) in plasma than LDL, which further increases the likelihood of atherosclerosis. Therefore, sdLDL has been identified as a risk factor for cardiovascular events by the National cholesterol Education Program [2]. Previous studies have shown that the size of sdLDL is positively correlated with esterification rates of fractional esterification rate of HDL-C (FER_HDL_) [3]. However, current methods for detecting sdLDL and FER_HDL_ have limitations, with time-consuming sample preparation and the requirements for radioisotopes make it difficult to promote detection in clinical laboratories[3].

Atherogenic index of plasma (AIP)-a logarithmic transition of the ratio of TG to HDL-C, is a new and better lipid marker in recent years, which was proposed by Dobiásová and Frohlich in 2001[4]. It was found that AIP was negatively correlated with lipoprotein particle size and FER_HDL_[4]. Previous studies have generally shown that individuals with dyslipidemia have a higher risk of developing T2D [5]. Triglycerides are the most abundant lipids in human adipose tissue. High levels of triglycerides can lead to lipotoxicity, which can contribute to the development and progression of IR [6, 7]. HDL-C contains hundreds of lipids and proteins that are known to play antioxidant and anti-inflammatory functions in the regulation of metabolic diseases, including diabetes [8, 9]. AIP combines TG and HDL-C levels and not only reflects the ratio of TG to HDL-C, but also represents the size of lipoprotein particles, which reflects the pathogenicity and specificity of dyslipidemia better than high TG or low HDL-C levels. [10].

AIP was initially constructed as a novel biomarker of plasma atherosclerosis to predict the risk of cardiovascular disease. In recent years, numerous studies have corroborated the relationship between the AIP and the risk of IR-related metabolic diseases, such as obesity [11, 12], prediabetes [13], diabetes [14, 15] and metabolic syndrome [16]. However, the relationship between AIP and IR has not been fully explored, only one clinical study reported that AIP can provide information on the severity of IR associated with impaired glucose metabolism in patients with T2D [17]. Herein, we conducted a nationally representative cross-sectional study based on the NHANES database to explore the non-linear association of AIP with IR and T2D in the general US population in search of a clinically accessible indicator for IR surveillance.

## Methods

### Data source and study sample

The National Health and Nutrition Examination Survey (NHANES) is a national survey of civilian non-institutional personnel in the United States conducted by the National Center for Health Statistics (NCHS) that using a cross-sectional, multistage, stratified, subgroup probability sampling study design with a two-year cycle [18]. The survey covers diverse aspects like face-to-face interviews at home (demographic, socioeconomic, dietary, and health-related questions), as well as health examinations (medical data) and anthropometry and laboratory tests collected at the Mobile Examination Center (MEC) [19, 20]. The NHANES protocol was revised and approved by the Ethics Review Committee of the NCHS, with all participants providing written informed consent [21].

**Fig. 1:**
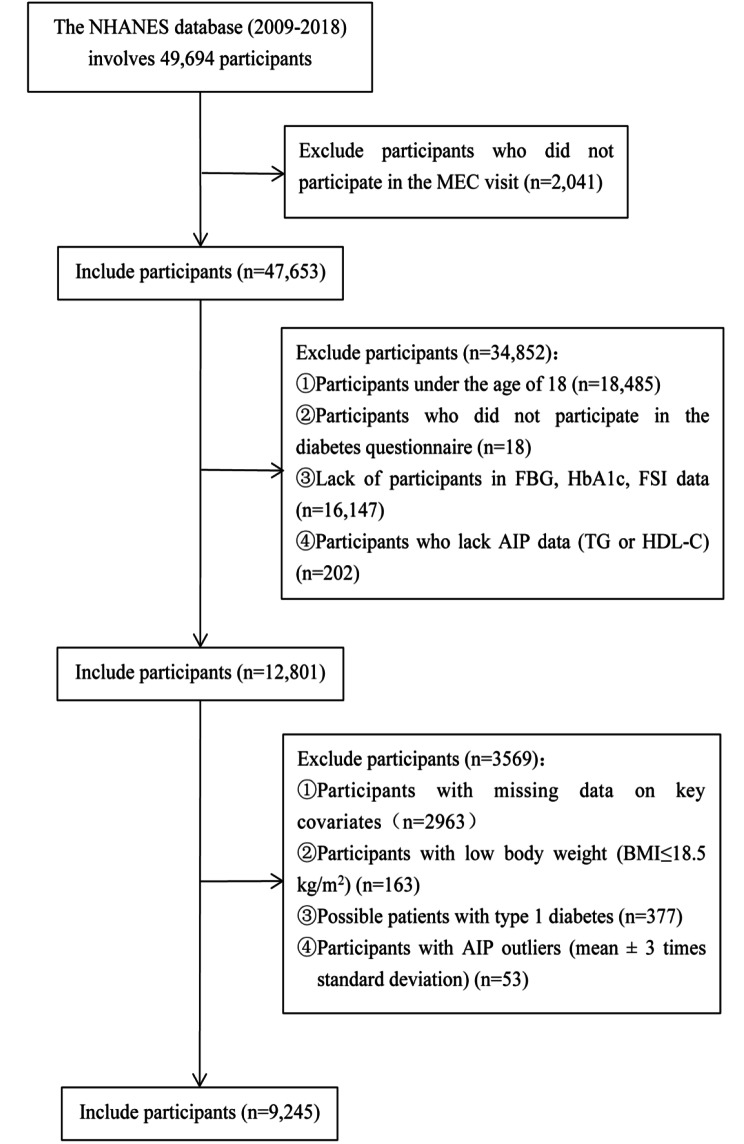
Flow chart of participants selection from the NHANES 2009–2018

The data for the population used in this cross-sectional study were all from from the NHANES database at five consecutive periods (2009–2010, 2011–2012, 2013–2014, 2015–2016 and 2017–2018), involving a total of 49,694 participants, which was consistent with the results of Wang et al. [22]. We excluded participants who have not attended a MEC visit, and we additionally excluded those who younger than 18 years of age, low body weight (BMI ≤ 18.5 kg/m^2^), possible individuals with type 1 diabetes (defined as those aged < 20 years who only receiving insulin treatment) or missing data (independent, dependent and covariant data) [23]. Besides, we also excluded participants with abnormal values of AIP (mean ± 3 times standard deviation). Finally, 9,245 participants (weighted n = 183,571,253) with complete data were included in this trial (Fig. 1).

### Exposure variable and outcome variables

The exposure variable was the AIP, which was mathematically derived from lg[TG(mmol/L)/HDL-C(mmol/L)] [4]. Subsequently, all participants were classified into four groups according to the AIP quartiles, including group Q1 (-1.01, -0.30), group Q2 (-0.30, -0.09), group Q3 (-0.09, 0.13), and group Q4 (0.13, 0.86). The outcome variables included the prevalence of IR and T2D and the risk markers of T2D, such as fasting blood glucose (FBG, mmol/L), glycosylated hemoglobin (HbA1c, %), fasting serum insulin (FSI, pmol/L) and homeostasis model assessment of insulin resistance (HOMA-IR). HOMA-IR was calculated with the formula: [FBG(mmol/L)*FSI(µU/ml)]/22.5 [24]. Referring to other studies, HOMA-IR > 2.6 is considered as IR of the normal population in the United States [25], which is used as a determination criterion in our study. In this study, according to relevant questionnaires and laboratory tests, T2D was defined as fasting plasma glucose (FPG) ≥ 7.0 mmol/L (126 mg/dL), oral glucose tolerance test 2 h plasma glucose ≥ 11.1 mmol/L (200 mg/dL), glycosylated hemoglobin (HbA1c) ≥ 6.5%, self-reported of T2D, or currently receiving hypoglycemic therapy [22, 26].

### Covariates

In this study, covariates were screened according to the following rules, (1) demographic data; (2) variables affecting AIP, IR, and T2D reported in previous studies; (3) according to the recommendations of the STROBE statement, the basic model changes by more than 10% after the introduction of covariates [27]; and (4) other variables accumulated in clinical experience.

Therefore, we included the following covariables that in line with the abovementioned rules: age, sex, race, education level, vigorous/moderate physical activity (V/MPA), smoking, alcohol consumption, systolic blood pressure (SBP, mmHg), diastolic blood pressure (DBP, mmHg), body mass index (BMI, kg/m^2^), waist circumference (WC, cm), total cholesterol (TC, mmol/L), alanine aminotransferase (ALT, U/L), gamma-glutamyl transpeptidase (γ-GGT, U/L), serum creatinine (SCr, umol/L), serum uric acid (SUA, umol/L), and Hemoglobi (Hb, g/dL).

Race/ethnicity was categorized as Mexican American, Other Hispanic, Non-Hispanic White, Non-Hispanic Black, or Other. Education level was categorized as below high school, high school, and above high school. Following the World Health Organization standards, general obesity was classified as normal weight (18.5 kg/m^2^ ≤ BMI < 25 kg/m^2^), overweight (25 kg/m^2^ ≤ BMI < 30 kg/m^2^) and obese (BMI ≥ 30 kg/m^2^) [28]. Central obesity was defined as WC > 102 cm for males or > 88 cm for females [29]. V/MPA was defined as having done at least 10 min of V/MPA in a typical week (2007–2018 cycle), resulting in significant sweating, or a substantial increase in respiration or heart rate [30]. Participants were divided into current smokers (average amount of smoking ≥ 1/day), never smokers (people who smoked less than 100 cigarettes in life) and former smokers (smoking > 100 cigarettes in life but currently did not smoke) [22]. Similarly, participants were classified as drinkers (more than 12 drinks a year) and non-drinkers (no more than 12 drinks a year) by drinking situation [31]. Referring to the American Heart Association Blood Pressure Guidelines 2017 [32], hypertension was usually defined as systolic blood pressure (SBP) ≥ 140mmHg and/or diastolic blood pressure (DBP) ≥ 90mmHg or self-reported hypertension and use of anti-hypertensive medication.

### Statistical analysis

Appropriate weighting methodology were used to account for complex sampling design to provide nationally representative results, as recommended by NHANES Guidelines [33].

AIP levels was divided into four groups based on quartiles (Q1: ≤ 25th percentile, Q2: > 25 to 50th percentile, Q3: > 50 to 75th percentile, Q4: > 75th percentile). The basic characteristics of categorical variables were expressed as counts and percentages (%), and the basic characteristics of continuous variables were described by means and standard deviation (SD) or median (interquartile range) (IQR). To analyze differences between continuous variables, a weighted linear regression model was used, while a weighted chi-square tests was performed to analyze differences between categorical variables.

Based on the STROBE statement [27], fully adjusted, minimally adjusted, and unadjusted, were applied in the present study (model 1: univariate logistic regression model; model 2: adjusted for age, gender, race, and education level; model 3: had additional adjustments for SBP, BMI, WC, smoking status, alcohol consumption, V/MPA, TC, ALT, γ-GGT, Cr, UA and Hb.

Three weighted multivariate linear regression model was developed to analyze the associations of AIP with the risk markers of T2D (FBG, HbA1c, FSI and HOMA-IR). Then, sensitivity analysis was performed to verify the stability of the results after excluding diabetic patients. To assess the relationships of AIP with IR and T2D, we used weighted multivariate logistic regression models and calculated odds ratios (ORs) and 95% CIs in three model. Subsequently, subgroup analysis were performed to test for interaction and control for confounding categorical variables, including age (≥ 40 or ≥ 40 and < 65 or ≥ 65), gender, race, education level, smoking status, alcohol consumption, V/MPA, general obesity, central obesity, and hypertension. Weighted multivariate logistic regression was used for the subgroup analysis. If the interaction P-value was not significant, then the results of the different strata are reliable, otherwise, there may be a special population.

We also examined the non-liner relationships of AIP with IR and T2D by generalized additive model (GAM) based on smooth curve fitting. When non-linearity was detected, a recursive algorithm was used to calculate the significant inflection points of the relationships of AIP with IR and T2D, and a threshold effect analysis was performed to assess the difference between the logistic regression model and the two-part logistic regression model.

All statistical analysis was performed using EmpowerStats (version 3.4.3, www.empowerstats.com) and R (version 4.2.2, http://www.R-project.org), and two-sided P < 0.05 indicated statistically significant.

## Results

### Characteristics of the participants

**Fig. 2:**
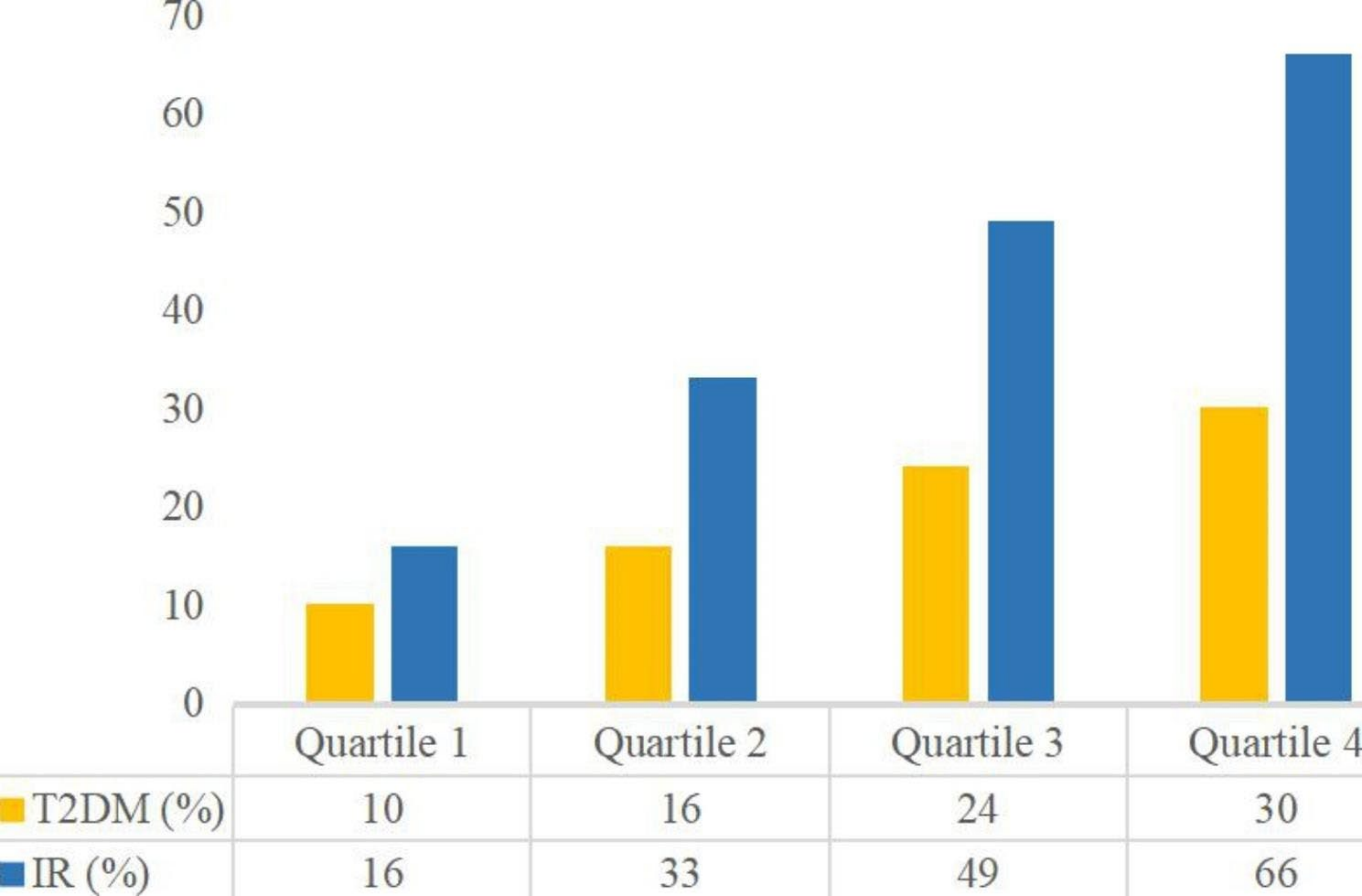
The proportions of T2D and IR by quartile of AIP

The baseline characteristics of the included participants according to AIP status are shown in Table 1. Compared with the participants in the lower AIP group, the participants in the AIP Q4 group were more male, more Mexican American, more current or former smoker, more people with lower education levels, more obesity or central obesity, and had higher levels of BMI, WC, SBP, DBP, FPG, HbA1c, FSI, HOMA-IR, TC, ALT, GGT, Cr, UA and Hb (all P < 0.05). Importantly, participants with high level s of AIP had higher prevalence of T2D and IR (all P < 0.05)  (Fig. 2).

**Table 1:** Characteristics of the study population from NHANES 2009–2018

Characteristics	AIP				*P* value
	Q1 (-1.01, -0.30)	Q2 (-0.30, -0.09)	Q3 (-0.09, 0.13)	Q4 (0.13, 0.86)	
Age (years)	45.70 ± 18.61	48.36 ± 18.38	50.36 ± 17.89	49.71 ± 16.28	< 0.0001
Age groups, n (%)					< 0.0001
Young age	969 (41.93)	836 (36.17)	720 (31.18)	700 (30.25)	
Middle age	891 (38.55)	952 (41.19)	985 (42.66)	1144 (49.44)	
Old age	451 (19.52)	523 (22.63)	604 (26.16)	470 (20.31)	
Gender, n (%)					< 0.0001
Male	850 (36.78)	1079 (46.69)	1238 (53.62)	1446 (62.49)	
Female	1461 (63.22)	1232 (53.31)	1071 (46.38)	868 (37.51)	
Race, n (%)					< 0.0001
Mexican American	226 (9.78)	343 (14.84)	406 (17.58)	467 (20.18)	
Other Hispanic	189 (8.18)	240 (10.39)	281 (12.17)	320 (13.83)	
Non-Hispanic White	875 (37.86)	948 (41.02)	962 (41.66)	1031 (44.55)	
Non-Hispanic Black	709 (30.68)	510 (22.07)	373 (16.15)	224 (9.68)	
Other Race	312 (13.50)	270 (11.68)	287 (12.43)	272 (11.75)	
Education levels, n(%)					< 0.0001
<high school	380 (16.44)	500 (21.64)	564 (24.43)	666 (28.78)	
=high school	496 (21.46)	497 (21.51)	552 (23.91)	552 (23.85)	
>high school	1435 (62.09)	1314 (56.86)	1193 (51.67)	1096 (47.36)	
Smoking status, n (%)					< 0.0001
Current smoker	333 (14.41)	399 (17.27)	460 (19.92)	576 (24.89)	
Former smoker	499 (21.59)	525 (22.72)	596 (25.81)	609 (26.32)	
Non-smoker	1479 (64.00)	1387 (60.02)	1253 (54.27)	1129 (48.79)	
Alcohol consumption, n (%)	1442 (63.52)	1466 (64.61)	1423 (62.66)	1454 (64.02)	0.0012
V/MPA, n (%)	1070 (47.14)	935 (41.21)	748 (32.94)	718 (31.62)	< 0.0001
WC (cm)	91.35 ± 15.23	97.75 ± 15.47	102.76 ± 15.59	106.55 ± 15.12	< 0.0001
Central obesity, n (%)	905 (39.16)	1259 (54.48)	1481 (64.14)	1608 (69.49)	< 0.0001
BMI (kg/m^2^)	26.57 ± 6.31	28.62 ± 6.65	30.32 ± 6.70	31.55 ± 6.55	< 0.0001
General obesity, n (%)					< 0.0001
Normal	1131 (48.94)	742 (32.11)	481 (20.83)	292 (12.62)	
Overweight	664 (28.73)	802 (34.70)	804 (34.82)	799 (34.53)	
Obesity	516 (22.33)	767 (33.19)	1024 (44.35)	1223 (52.85)	
Blood pressure					
SBP (mmHg)	119.47 ± 17.59	121.84 ± 18.48	123.58 ± 17.89	124.62 ± 17.21	< 0.0001
DBP (mmHg)	67.17 ± 12.64	68.18 ± 13.50	68.96 ± 13.70	70.98 ± 13.27	< 0.0001
Laboratory data					
FBG (mmol/L)	5.53 ± 1.15	5.80 ± 1.48	6.15 ± 1.91	6.61 ± 2.43	< 0.0001
HBA1C (%)	5.50 ± 0.68	5.65 ± 0.93	5.84 ± 1.14	6.04 ± 1.32	< 0.0001
FSI (pmol/L)	39.54 (27.21,59.52)	53.40 (35.82,80.37)	67.68 (43.92,103.56)	88.50 (59.34,137.37)	< 0.0001
HOMA-IR	1.36 (0.92,2.11)	1.91 (1.23,3.03)	2.55 (1.58,4.12)	3.53 (2.25,5.78)	< 0.0001
TC (mmol/L)	4.68 ± 0.95	4.82 ± 1.00	4.93 ± 1.06	5.22 ± 1.08	< 0.0001
ALT (U/L)	18.00 (14.00,23.00)	19.00 (15.00,26.00)	22.00 (16.00,29.00)	25.00 (19.00,35.00)	< 0.0001
γ-GGT (U/L)	16.00 (12.00,23.00)	18.00 (13.00,26.00)	20.00 (15.00,31.00)	25.50 (18.00,39.00)	< 0.0001
SCr (umol/L)	70.72 (61.00,83.10)	73.37 (61.88,87.52)	75.14 (62.76,89.28)	77.79 (64.53,90.17)	< 0.0001
SUA (umol/L)	291.66 ± 74.13	315.89 ± 78.36	338.81 ± 83.52	361.49 ± 86.22	< 0.0001
Hb (g/dL)	13.65 ± 1.47	14.02 ± 1.47	14.27 ± 1.49	14.54 ± 1.49	< 0.0001

*AIP* atherogenic index of plasma, *V/MPA* vigorous/moderate physical activityvigorous/moderate physical activity, *WC* waist circumference, *BMI* body mass index, *SBP* systolic blood pressure, *DBP* diastolic blood pressure, *FPG* fasting plasma glucose, *HbA1c* glycosylated hemoglobin, *FSI* fasting serum insulin, *HOMA-IR* homeostasis model assessment of insulin resistance, *TC* total cholesterol, *ALT* alanine aminotransferase, *γ-GGT* gamma-glutamyl transpeptidase, *SCr* serum creatinine, *SUA* serum uric acid, *Hb* hemoglobi.

Mean ± SD and Median (IQR) for continuous variables, *P* value was calculated by weighted linear regression model. % for categorical variables, *P* value was calculated by weighted chi-square test.

### Association between AIP and risk markers of T2D

Table 2 shows the association between AIP and risk markers for T2D. In the fully adjusted multivariate linear regression models, the associations between AIP and FPG (β = 0.08, 95% CI: 0.06, 0.10), HbA1c (β = 0.04, 95% CI: 0.39, 0.58), FSI (β = 4.26, 95% CI: 3.73, 4.79), and HOMA-IR (β = 0.22, 95% CI: 0.18, 0.25) were significantly positive. The trend remained to be of statistical significance among the AIP quartile groups, with participants in the second, third, and fourth quartiles of AIP having progressively higher levels of FPG, HbA1c, FSI, and HOMA-IR compared to the lowest quartile (all P for Trend < 0.0001). In sensitivity analyses, this positive association was still observed in the fully adjusted model after excluding 1,858 diabetic patients (see Additional file 1: Table S1).

**Table 2:** The associations between AIP and risk markers of T2D

Outcomes	β (95% CI)					*P* for trend
	AIP * 10	Q1 (-1.01, -0.30)	Q2 (-0.30, -0.09)	Q3 (-0.09, 0.13)	Q4 (0.13, 0.86)	
FBG						
Model 1	0.12 (0.10, 0.144)	Reference	0.22 (0.14, 0.29)	0.45 (0.37, 0.53)	0.99 (0.84, 1.13)	< 0.0001
Model 2	0.11 (0.09, 0.12)	Reference	0.17 (0.09, 0.25)	0.34 (0.25, 0.42)	0.86 (0.72, 1.00)	< 0.0001
Model 3	0.08 (0.06, 0.10)	Reference	0.07 (-0.01, 0.16)	0.18 (0.09, 0.27)	0.63 (0.49, 0.78)	< 0.0001
HBA1C						
Model 1	0.06 (0.05, 0.07)	Reference	0.13 (0.08, 0.17)	0.25 (0.21, 0.30)	0.51 (0.43, 0.59)	< 0.0001
Model 2	0.06 (0.05, 0.07)	Reference	0.11 (0.07, 0.15)	0.21 (0.16, 0.26)	0.47 (0.39, 0.55)	< 0.0001
Model 3	0.04 (0.03, 0.05)	Reference	0.06 (0.02, 0.11)	0.13 (0.08, 0.18)	0.36 (0.29, 0.43)	< 0.0001
FSI						
Model 1	8.09(7.38, 8.79)	Reference	18.34 (14.64, 22.05)	37.34(31.20, 43.47)	66.73(59.92, 73.55)	< 0.0001
Model 2	8.30(7.61, 8.98)	Reference	18.82 (15.08, 22.56)	38.01(31.91, 44.11)	67.87(61.19, 74.56)	< 0.0001
Model 3	4.26(3.73, 4.79)	Reference	4.75 (0.99, 8.50)	13.77 (8.03, 19.52)	34.68(29.98, 39.38)	< 0.0001
HOMA-IR						
Model 1	0.40 (0.35, 0.44)	Reference	0.79 (0.57, 1.02)	1.68 (1.32, 2.04)	3.25 (2.84, 3.65)	< 0.0001
Model 2	0.39 (0.35, 0.44)	Reference	0.78 (0.55, 1.01)	1.63 (1.25, 2.01)	3.20 (2.81, 3.60)	< 0.0001
Model 3	0.22 (0.18, 0.25)	Reference	0.16 (-0.09, 0.41)	0.56 (0.19, 0.93)	1.75 (1.43, 2.06)	< 0.0001

### Relationship between AIP and T2D

Regardless of whether the confounding variables were adjusted for, the associations between AIP and the prevalence of T2D were positive in all multivariable logistic regression models (model 1: OR = 1.20, 95% CI: 1.17–1.22; model 2: OR = 1.23, 95% CI: 1.19–1.26; model 3: OR = 1.18, 95% CI: 1.15–1.22). Notably, after adjusting for potential confounding variables (model 3), each 0.1-unit increase in AIP was associated with an 18% increase in the odds of T2D. Moreover, this trend remained statistically significant when AIP was considered a categorical variable (quartiles), with a progressively higher risk of T2D in the quartile with higher AIP compared with the lowest quartile in all multivariate logistic regression models (*P* for trend < 0.0001). The results are presented in Table 3.

**Table 3:** The associations between AIP and T2D

AIP	Model 1		Model 2		Model 3	
	OR (95%CI)	*P* value	OR (95%CI)	*P* value	OR (95%CI)	*P* value
AIP * 10	1.20 (1.17, 1.22)	< 0.0001	1.23 (1.19, 1.26)	< 0.0001	1.18 (1.15, 1.22)	< 0.0001
AIP (Quartile )						
Q1(-1.01, -0.30)	Reference		Reference		Reference	
Q2(-0.30, -0.09)	2.00 (1.60, 2.50)	< 0.0001	2.03 (1.58, 2.62)	< 0.0001	1.77 (1.35, 2.31)	0.0001
Q3(-0.09, 0.13)	3.25 (2.66, 3.97)	< 0.0001	3.22 (2.54, 4.08)	< 0.0001	2.44 (1.88, 3.16)	< 0.0001
Q4(0.13, 0.86)	5.10 (4.18, 6.22)	< 0.0001	5.85 (4.56, 7.51)	< 0.0001	4.34 (3.36, 5.61)	< 0.0001
*P* for trend	< 0.0001		< 0.0001		< 0.001	

In the sub-analyses stratified by age, gender, race, education level, smoking status, alcohol consumption, V/MPA, general obesity, central obesity, and hypertension, the association between AIP and the prevalence of T2D was stable (all P < 0.05). However, gender was considered the most prominent interactive factor influencing the relationship between AIP and T2D (P for interaction = 0.0024). With the increase in the AIP, the risk of T2D among female was more significant than that among male (OR = 11.98, 95% CI: 7.94–18.08). See Additional file 1: Table S2 and Fig.S1 for details.

### Relationship between AIP and IR

An increased AIP level was related to the elevated risk of IR in all multivariate logistic regression models (model 1: OR = 1.20, 95% CI: 1.32–1.38; model 2: OR = 1.37, 95% CI: 1.34–1.41; model 3: OR = 1.29, 95% CI: 1.26–1.32). Notably, after adjusting for potential confounding variables (model 3), each 0.1-unit increase in AIP was associated with an 29% increase in the odds of IR. Moreover, this trend remained statistically significant when AIP was considered a categorical variable (quartiles), with a progressively higher risk of IR in the quartile with higher AIP compared with the lowest quartile in all multivariate logistic regression models (P for trend < 0.0001). The results are presented in Table 4.

**Table 4:** The associations between AIP and IR

AIP	Model 1		Model 2		Model 3	
	OR (95%CI)	*P* value	OR (95%CI)	*P* value	OR (95%CI)	*P* value
AIP * 10	1.35 (1.32, 1.38)	< 0.0001	1.37 (1.34, 1.41)	< 0.0001	1.29(1.26, 1.32)	< 0.0001
AIP (Quartile )						
Q1(-1.01,-0.30)	Reference		Reference		Reference	
Q2(-0.30,-0.09)	3.22 (2.41, 4.30)	< 0.0001	3.33 (2.49, 4.46)	0.0001	2.47 (1.71, 3.56)	< 0.0001
Q3(-0.09, 0.13)	5.95 (4.56, 7.75)	< 0.0001	6.28 (4.78, 8.25)	< 0.0001	3.61 (2.58, 5.06)	< 0.0001
Q4(0.13, 0.86)	14.01 (10.65, 18.44)	< 0.0001	15.51 (11.58, 20.76)	< 0.0001	8.27 (6.08, 11.26)	< 0.0001
*P* for trend	< 0.0001		< 0.0001		< 0.001	

In the sub-analyses stratified by age, gender, race, education level, smoking status, alcohol consumption, V/MPA, general obesity, central obesity, and hypertension, the association between AIP and the prevalence of IR was stable (all P < 0.05). However, gender (P for interaction = 0.0135) and smoking (P for interaction = 0.0130) were considered the most prominent interactive factor influencing the relationship between AIP and IR. With the increase in the AIP, the risk of IR among female was more significant than that among male (OR = 17.03, 95% CI: 11.21–25.86). Participants who did not smoke had a stronger association between AIP and risk of IR than those who smoked in the past and now (OR = 17.98, 95% CI: 11.74–27.55). The results are presented in Additional file 1: Table S3 and Fig.S2.

### Non-linear relationships

**Fig. 3:**
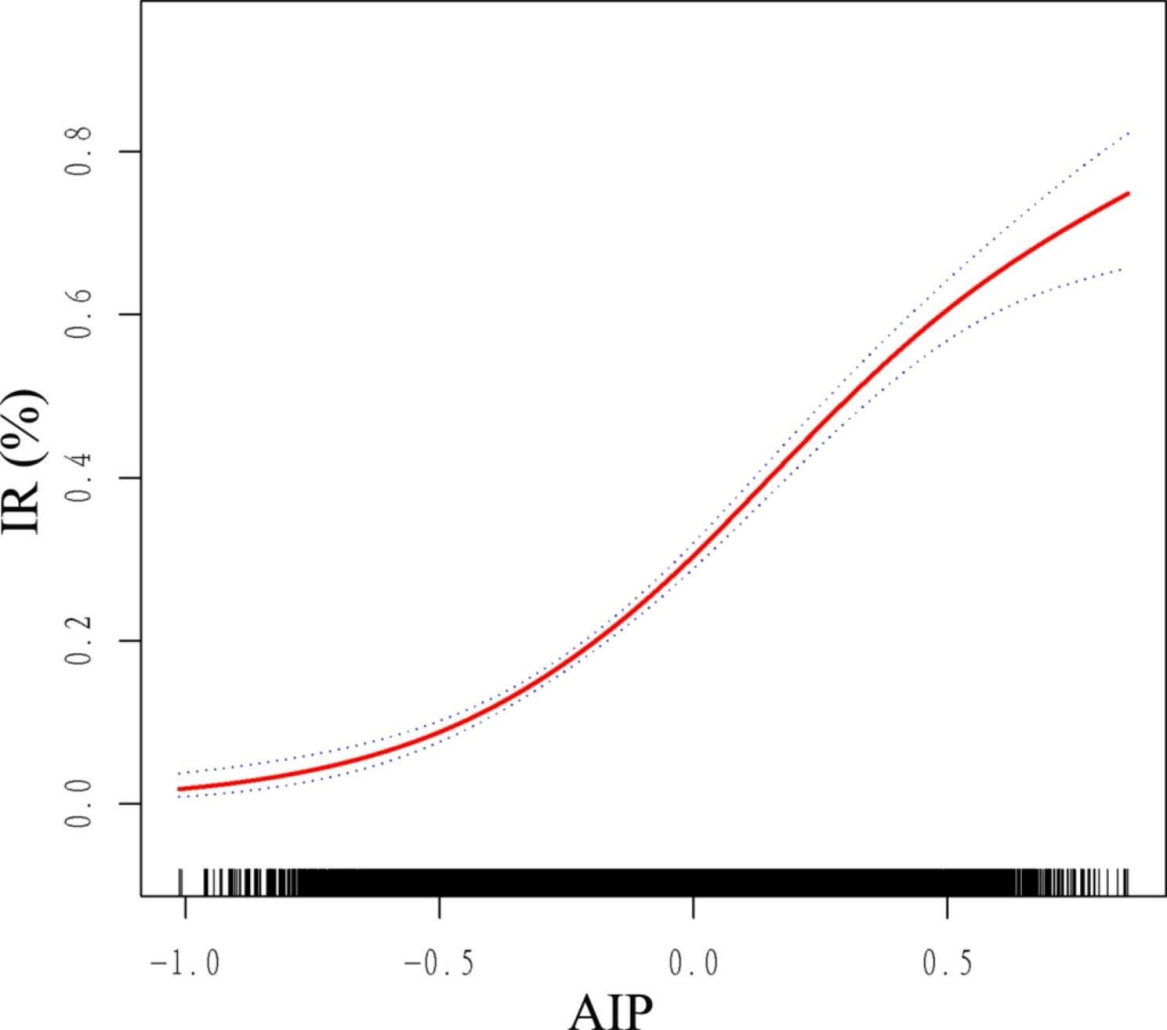
Smooth curve fitting using GAM to evaluate the nonlinear relationship between AIP and the risk of IR. The red solid line represents the probability of IR occurrence and the blue dotted line represents the 95% CI curve

Here, we performed a GAM and a smooth curve fitting to detect the non-linear relationships of AIP with risk of IR and T2D and further confirm the results. In the fully adjusted model, a non-linear and reverse L-shaped association was detected between AIP and IR (Fig. 3), with a inflection point of 0.45 by threshold effect analysis (Table 5). It was found that there was a significant positive correlation between AIP and IR before the inflection point, and the OR (95%CI) was 13.15 (10.45,16.55). However, the association between AIP and IR was not significant after the inflection point, with the OR (95% CI) being 0.67 (0.12, 3.85).

**Table 5:** Threshold effect analysis of AIP on IR using a two-part logistic regression model

AIP	Adjusted OR (95% CI), *P* value
model I	
Fitting by the standard linear model	11.43 (9.25, 14.13), < 0.0001
model II	
Inflection point	0.45
< 0.45	13.15 (10.45, 16.55), < 0.0001
> 0.45	0.67 (0.12, 3.85), 0.6567
Log likelihood ratio	0.002

**Fig. 4:**
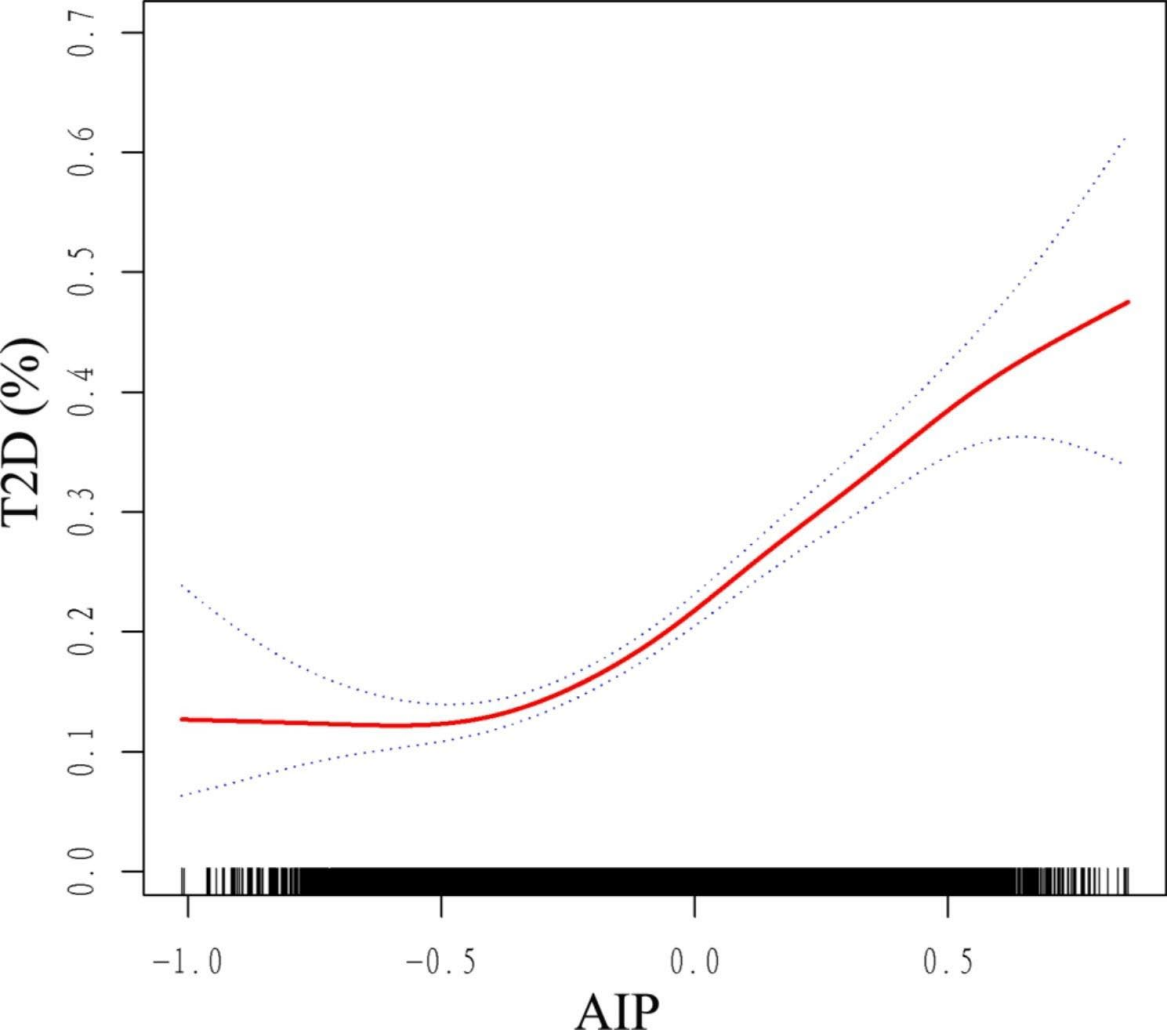
Smooth curve fitting using GAM to evaluate the nonlinear relationship between AIP and the risk of T2D. The red solid line represents the probability of T2D occurrence and the blue dotted line represents the 95% CI curve

Furthermore, a non-linear and J-type association was detected between AIP and T2D, with a inflection point of -0.47 by threshold effect analysis (Fig. 4; Table 6). When AIP was >-0.47, increased AIP was significantly associated with increased risk of T2D (OR = 5.39, 95% CI: 4.21, 6.89). But there was no significant association between AIP and T2D when AIP <-0.47 (OR = 0.30, 95% CI: 0.07, 1.35).

**Table 6:** Threshold effect analysis of AIP on T2D using a two-part logistic regression model

AIP	Adjusted OR (95% CI), *P* value
model I	
Fitting by the standard linear model	4.58 (3.64, 5.74), < 0.0001
model II	
Inflection point	-0.47
< -0.47	0.30 (0.07, 1.35), 0.1166
> -0.47	5.39 (4.21, 6.89), < 0.0001
Log likelihood ratio	< 0.001

In the subgroup analysis stratified by age, gender, race, education level, smoking status, alcohol consumption, V/MPA, general obesity, central obesity, and hypertension, the non-linear relationships between AIP and the risk of IR and T2D still existed (Additional file 1: Fig. S3, Fig. S4).

## Discussion

In this study of US adults, we found a positive associations between AIP and risk markers for T2D after adjustment for confounders. We further found that AIP was associated with an increased risk of developing IR and T2D. However, the positive associations between AIP and IR or T2D were more significant in female than in male. Moreover, a reverse L-shaped curve with an inflection point of 0.45 was detected between AIP and IR. Furthermore, a J-type curve with an inflection point of -0.47 was detected between AIP and T2D. These findings indicate that AIP has the potential to be used as a monitoring indicator of IR and T2D.

Dobiásová et al. showed the skewed distribution of TG/HDL-C ratio, and the logarithmic transformation can be used to approximate the normal distribution [4], as we found when dealing with the original data. As can be seen from Table 1, some traditional risk factors for diabetes, such as older age, smoking, higher WC, BMI and blood pressure are more likely to have higher AIP values, while protective factor such as V/MPA are more likely to have lower AIP values. Our study shows that AIP is positively correlated with and FPG, HbA1c, FSI and HOMA-IR, which is an independent risk factor for the occurrence of IR and T2D. Tan et al. found that AIP was significantly negatively correlated with HOMA-IR but no significant negative correlation with HbA1c which was inconsistent with our findings [17]. Considering that the data for this study came from a randomized, double-blind, multicenter, placebo-controlled clinical trial of patients with T2D, higher AIP values at baseline may have influenced the results. Two prospective cohort studies in China suggest that AIP was significantly associated with T2D in middle-aged and older adults [14, 16]. An 8-year prospective cohort study of the Turkish population also found that AIP independently predicted obesity-mediated T2D in men and women [34].

IR may vary by age, gender, race, V/MPA, BMI, WC, blood pressure, smoking and alcohol consumption status, etc. A large cohort study of multi-ethnic obese adolescents in the US showed that the association between TG/HDL-C ratio and IR varied by race, with no association found in Hispanic and African-American individuals [35]. Whereas another cross-sectional study involving 99 African Americans and 50 whites aged 18–45 years demonstrated that the relationship between TG/HDL-C and IR varied by race and that it was inappropriate to use TG/HDL-C to predict IR in African Americans [36]. Further studies found that TG/HDL-C did not recognize IR in African American women [37]. Our study further validated the association of AIP with T2D and IR in subgroups and found that the influence of AIP on the risk of IR and T2D was stable across subgroups except for gender and smoking status (P < 0.05). In conclusion, our findings imply that higher AIP can be an important predictor of IR and T2D.

Our study found that the positive correlation between AIP and IR or T2D was more pronounced in women than in men, although male subjects were more likely to have higher AIP values, and smooth curve fitting also revealed different curve shapes for AIP and T2D risk in men and women. This is similar to a case-control study in the Singapore Chinese Health Study [38]. In addition, studies of the relationship between TG/HDL-C ratio and IR and T2D in Chinese, Korean and Iranian populations also support the existence of gender differences [39–42]. A cross-sectional study in the US suggested that the TG/HDL-C ratio could be used to identify IR in Hispanics and African Americans, while the association with T2D was only found in females [43]. The underlying mechanisms of the observed gender differences are unclear. Although previous studies have found that women exhibit more favorable metabolic risk profiles than men, including lower TG and higher HDL-C levels [44], sex chromosome differences and postmenopausal decreases in estrogen levels promote disturbances in glucose and lipid metabolism, so it is possible that women are at higher risk for T2D [45]. Previous studies have generally concluded that the association between dyslipidemia and diabetes appears to be stronger in smokers. Conversely, in our study, the association between AIP and IR was significantly higher in non-smokers than in smokers (both current and former smokers). This is similar to the results of a large multicenter clinical study entitled “Retrospective cohort study on the adjuvant treatment of angina pectoris in coronary heart disease with proprietary Chinese medicines (RCSCD-TCM)” in China, in which the association between TG/HDL-C ratio and T2D was stronger in non-smokers with CHD when TG/HDL-C was a continuous variable [46]. The reason for this phenomenon may still be related to gender differences as the non-smoking population is predominantly females, which may need to be verified by further longitudinal studies.

Certainly, some of the results of this study differ from previous studies. Our study found that the correlation of AIP with IR and T2D was different on different sides of the inflection point when fully adjusted for potential confounders, with the risk of T2D increasing with increasing AIP levels when he AIP was greater than − 0.47, and the risk of IR increasing with increasing AIP levels when the AIP was below 0.45. Notably, when AIP started to rise, it is positively correlated with IR but not with the risk of T2D, and when AIP rises to a certain value (AIP=-0.47), it is positively correlated with both IR and T2D. when AIP continued to rise to a certain height (AIP = 0.45), it was no longer correlated with IR, but still positively correlated with T2D. These findings suggest that early clinical blood lipid intervention is necessary to prevent IR in the early stage of dyslipidemia to prevent further development of T2D.

### Limitations and strengths

The main strength of this study is its large sample size. The NHANES database uses a complex weighted design that is more representative of the entire US population, whereas we used data from NHANES from 2009 to 2018. Second, the definition of T2D in this study included not only a self-reported history of T2D and/or FPG ≥ 7.0 mmol/L, but also a 2-hour oral glucose tolerance test and HbA1c measurement, thus minimizing missed diagnoses. Additionally, NHANES uses standardized procedures for data collection by professional and trained personnel, including a standard questionnaire, physical examination, and laboratory tests for each participant.All blood samples are tested in the same laboratory using standard protocols in the same cycle, which greatly reduces potential bias due to different methods. Moreover, a stratified analysis of the association of AIP with IR and T2D was performed across age stage, gender, race, education level, smoking and drinking status to explore the impact of potential confounders on the association and to improve statistical power. In addition, we used smoothed fitting curves and two-part logistic regression to accurately analyze the nonlinear relationships for the first time.

However, some limitations of the present study should be noted. First, this is an observational study in which causality cannot be determined, and we should interpret the present findings carefully, as both causal and reverse causal explanations are possible. Therefore, further prospective studies are needed to determine the exact relationship between AIP and the risk of IR and T2D. Second, although we have controlled and adjusted for a range of covariates, there may still be potential confounders that were not measured, such as dietary patterns and family history of T2D. Circulating TG levels are strongly influenced by diet. However, blood samples were collected after fasting, which may have improved the results, but further studies are needed to determine the effect of diet on study outcomes. Furthermore, some investigations suggest that longitudinal changes in AIP may have potential predictive value for T2D [14]. Hence, further research is needed in the future to investigate the role of the combination of baseline and variability in AIP in the development of IR and T2D.

## Conclusion

Collectively, an inverse L-shaped association was found between AIP and IR, while a J-shaped association was found between AIP and T2D. In patients with − 0.47 < AIP < 0.45, higher AIP was significantly associated with higher risk of IR and T2D. These findings suggest that reducing AIP levels within a certain range may have a positive effect on the prevention and treatment of IR and T2D. However, more investigations are still needed to confirm the causal relationship and underlying mechanisms.

## Electronic supplementary material

Below is the link to the electronic supplementary material.

Supplementary Material 1

## Data Availability

Publicly available datasets were analyzed in this study. This data can be found in NHANES’s official website, at http://www.cdc.gov/nchs/nhanes.htm.
