# Supplementary material for: Non-linear association of atherogenic index of plasma with insulin resistance and type 2 diabetes: a cross-sectional study
Source: Cardiovasc Diabetol. 2023 Jun 29;22:157. doi: 10.1186/s12933-023-01886-5 (PMC10311747; doi:10.1186/s12933-023-01886-5)
Supplement: Supplementary file 1 — Supplementary Material 1 [file 12933_2023_1886_MOESM1_ESM.docx]

Supplementary Material

**Table S1 The association between AIP and the markers of T2D risk markers (sensitivity analysis)**

| Outcomes | β (95% CI) | | | | | *P* for trend |
| --- | --- | --- | --- | --- | --- | --- |
|  | Per SD increase | Q1 (−1.01, −0.30) | Q2 (-0.30, -0.09) | Q3 (-0.09, 0.13) | Q4 (0.13, 0.86) |  |
| FBG | 0.10 (0.03, 0.17) | Reference | -0.00 (-0.05, 0.04) | 0.02 (-0.02, 0.07) | 0.07 (0.02, 0.13) | 0.0101 |
| HbA1C | 0.10 (0.06, 0.15) | Reference | 0.03 (0.01, 0.06) | 0.05 (0.02, 0.07) | 0.08 (0.04, 0.11) | <0.0001 |
| FSI | 42.38 (36.69, 48.07) | Reference | 4.30 (1.31, 7.29) | 12.87 (9.39, 16.35) | 31.29 (26.84, 35.74) | <0.0001 |
| HOMA-IR | 1.53 (1.31, 1.76) | Reference | 0.12 (-0.00, 0.24) | 0.44 (0.30, 0.58) | 1.13 (0.95, 1.31) | <0.0001 |

CI, confidence intervals

SD, standard deviation.

Adjusted for age (continuous), gender (categorical), race (categorical), education level (categorical), smoking status (categorical), alcohol consumption (categorical), V/MPA (categorical), BMI (continuous), WC (continuous), SBP (continuous), DBP (continuous), TC (continuous), ALT (continuous), Cr (continuous), UA (continuous), γ-GGT (continuous), HB (continuous).

1,858 participants with diabetes were excluded.

**Table S2 Subgroup analysis of the associations between AIP and T2D.**

| AIP | Total | OR(95%CI) | | | | *P* for trend | *P* for interaction |
| --- | --- | --- | --- | --- | --- | --- | --- |
|  |  | Q1 (−1.01, −0.30) | Q2 (-0.30, -0.09) | Q3 (-0.09, 0.13) | Q4 (0.13, 0.86) |  |  |
| Gender |  |  |  |  |  |  | 0.0024 |
| Male | 4.78 (3.12, 7.32) | Reference | 1.31(0.87,1.97) | 1.54 (1.08, 2.21) | 2.93 (2.10, 4.09) | <0.0001 |  |
| Female | 10.94 (6.79, 17.62) | Reference | 2.24 (1.61, 3.14) | 3.59 (2.55, 5.04) | 6.15 (4.21, 9.00) | <0.0001 |  |
| Age |  |  |  |  |  |  | 0.3322 |
| Young age | 6.74 (3.00, 15.17) | Reference | 1.48 (0.58, 3.77) | 1.69 (0.77, 3.75) | 3.82 (1.93, 7.57) | <0.0001 |  |
| Middle age | 8.27 (5.08, 13.44) | Reference | 1.80 (1.19, 2.73) | 2.43 (1.71, 3.46) | 4.75 (3.18, 7.10) | <0.0001 |  |
| Old age | 4.77 (2.97, 7.66) | Reference | 1.80 (1.15, 2.80) | 2.55 (1.67, 3.90) | 3.25 (2.26, 4.67) | <0.0001 |  |
| Race |  |  |  |  |  |  | 0.3994 |
| Mexican American | 12.77 (4.81, 33.90) | Reference | 1.72 (0.74, 3.99) | 2.32 (1.02, 5.25) | 5.79 (2.28, 14.69) | <0.0001 |  |
| Other Hispanic | 4.38 (1.61, 11.93) | Reference | 1.04 (0.47, 2.34) | 1.46 (0.68, 3.12) | 2.50 (1.14, 5.44) | 0.0057 |  |
| Non-Hispanic White | 7.58 (4.78, 12.02) | Reference | 2.06 (1.34, 3.17) | 2.69 (1.81, 4.00) | 4.82 (3.24, 7.17) | <0.0001 |  |
| Non-Hispanic Black | 5.13 (3.14, 8.40) | Reference | 1.70 (1.17, 2.46) | 2.64 (1.72, 4.03) | 3.00 (2.09, 4.32) | <0.0001 |  |
| Other Race | 6.15 (2.70, 14.02) | Reference | 0.93 (0.45, 1.91) | 1.87 (1.01, 3.47) | 3.16 (1.71, 5.82) | 0.0001 |  |
| Education levels |  |  |  |  |  |  | 0.0678 |
| <high school | 6.33 (3.27, 12.25) | Reference | 1.12 (0.59, 2.12) | 2.63 (1.38, 5.00) | 3.31 (1.88, 5.84) | <0.0001 |  |
| =high school | 8.84 (4.65, 16.80) | Reference | 2.33 (1.35, 4.01) | 2.22 (1.32, 3.76) | 5.54 (3.34, 9.20) | <0.0001 |  |
| >high school | 6.85 (4.46, 10.54) | Reference | 1.80 (1.31, 2.50) | 2.43 (1.74, 3.40) | 4.25 (3.06, 5.89) | <0.0001 |  |
| Smoking |  |  |  |  |  |  | 0.3878 |
| Current smoker | 7.95 (3.49, 18.09) | Reference | 1.17 (0.52, 2.67) | 1.77 (0.91, 3.45) | 3.76 (1.97, 7.19) | <0.0001 |  |
| Former smoker | 8.52 (4.27, 17.03) | Reference | 1.96 (1.17, 3.31) | 2.30 (1.43, 3.67) | 5.06 (2.88, 8.89) | <0.0001 |  |
| Non-smoker | 6.30 (4.47, 8.89) | Reference | 1.82 (1.27, 2.61) | 2.73 (2.00, 3.73) | 4.00 (3.00, 5.33) | <0.0001 |  |
| Alcohol consumption |  |  |  |  |  |  | 0.9712 |
| No | 7.72 (4.63, 12.87) | Reference | 1.84 (1.26, 2.70) | 2.62 (1.74, 3.94) | 4.61 (3.06, 6.95) | <0.0001 |  |
| Yes | 6.90 (4.66, 10.22) | Reference | 1.72 (1.19, 2.48) | 2.33 (1.69, 3.20) | 4.18 (3.07, 5.70) | <0.0001 |  |
| V/MPA |  |  |  |  |  |  | 0.6315 |
| No | 6.74 (4.71, 9.65) | Reference | 1.87 (1.43, 2.44) | 2.58 (1.94, 3.42) | 4.27 (3.24, 5.61) | <0.0001 |  |
| Yes | 8.41 (5.02, 14.09) | Reference | 1.53 (0.86, 2.72) | 2.09 (1.28, 3.43) | 4.52 (2.91, 7.02) | <0.0001 |  |
| General obesity |  |  |  |  |  |  | 0.5491 |
| Normal | 7.37 (3.42, 15.93) | Reference | 1.85 (1.22, 2.82) | 2.73 (1.58, 4.70) | 4.23 (2.37, 7.55) | <0.0001 |  |
| Overweight | 7.54 (4.34, 13.09) | Reference | 1.36 (0.88, 2.11) | 2.49 (1.61, 3.85) | 4.00 (2.65, 6.04) | <0.0001 |  |
| Obesity | 2.03 (1.27, 3.23) | Reference | 1.92 (1.23, 3.00) | 2.28 (1.48, 3.51) | 4.40 (2.98, 6.49) | <0.0001 |  |
| Abdominal obesity |  |  |  |  |  |  | 0.2748 |
| No | 6.20 (3.38, 11.36) | Reference | 1.49 (0.98, 2.28) | 2.67 (1.73, 4.13) | 3.58 (2.24, 5.72) | <0.0001 |  |
| Yes | 8.16 (5.69, 11.71) | Reference | 1.91 (1.38, 2.64) | 2.51 (1.85, 3.41) | 4.87 (3.59, 6.60) | <0.0001 |  |
| Hypertension |  |  |  |  |  |  | 0.6700 |
| No | 7.20 (4.03, 12.87) | Reference | 1.57 (1.04, 2.38) | 2.05 (1.35, 3.13) | 4.16 (2.67, 6.48) | <0.0001 |  |
| Yes | 6.67 (4.69, 9.49) | Reference | 1.84 (1.34, 2.54) | 2.62 (1.92, 3.58) | 4.23 (3.25, 5.52) | <0.0001 |  |


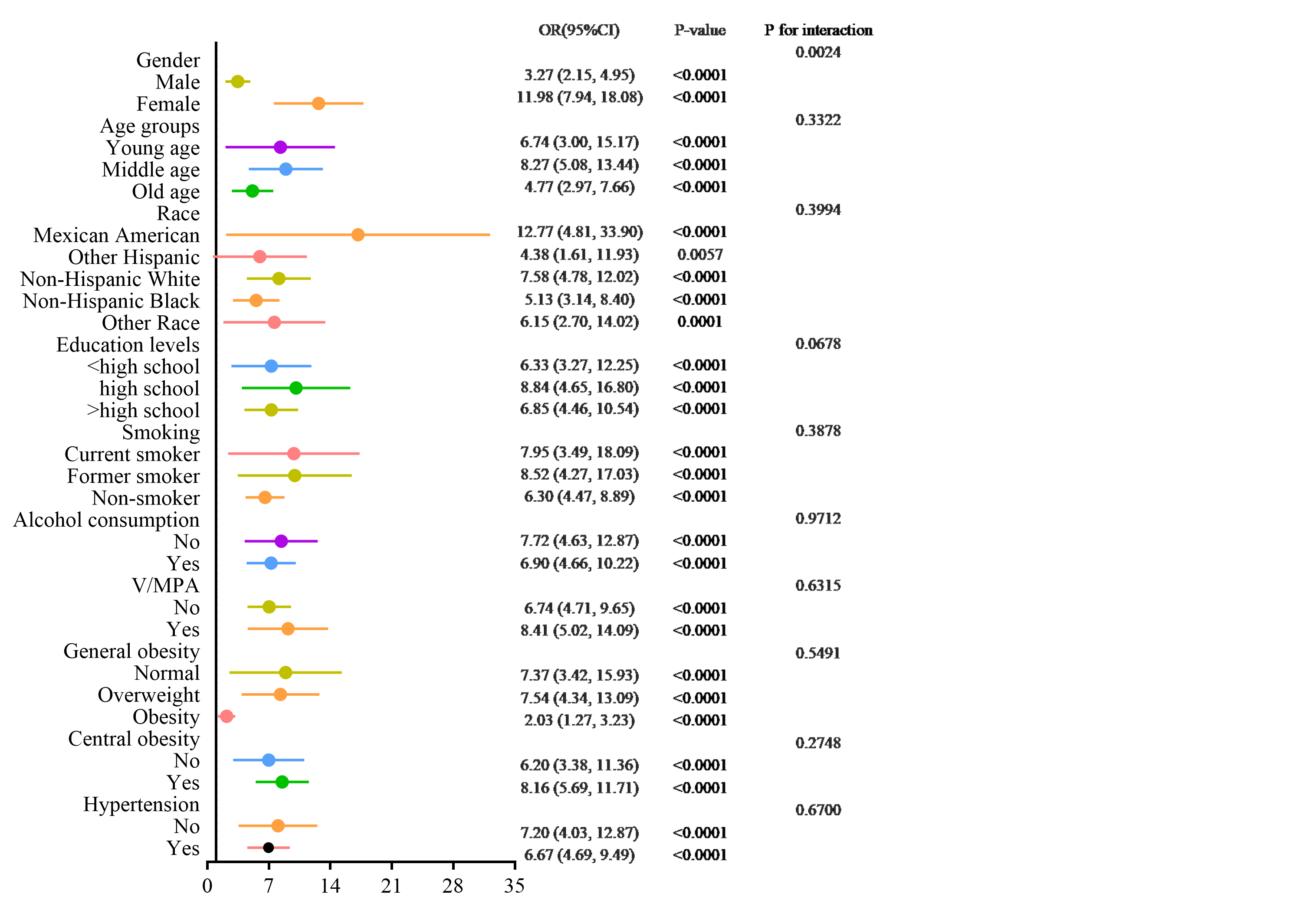


**Fig. S1** Subgroup analysis of AIP and T2D

**Table S3 Subgroup analysis of the associations between AIP and IR.**

| AIP | Total | OR(95%CI) | | | | *P* for trend | *P* for interaction |
| --- | --- | --- | --- | --- | --- | --- | --- |
|  |  | Q1 (−1.01, −0.30) | Q2 (-0.30, -0.09) | Q3 (-0.09, 0.13) | Q4 (0.13, 0.86) |  |  |
| Gender |  |  |  |  |  |  | 0.0135 |
| Male | 9.39 (6.39, 13.82) | Reference | 1.57 (1.14, 2.17) | 2.82 (2.14, 3.72) | 4.86 (3.61, 6.56) | <0.0001 |  |
| Female | 17.03 (11.21, 25.86) | Reference | 2.58 (1.90, 3.52) | 3.99 (2.96, 5.38) | 8.44 (6.15, 11.58) | <0.0001 |  |
| Age |  |  |  |  |  |  | 0.2360 |
| Young age | 11.43 (7.04, 18.54) | Reference | 1.76 (1.19, 2.61) | 3.55 (2.42, 5.23) | 5.60 (3.86, 8.12) | <0.0001 |  |
| Middle age | 14.50 (9.22, 22.81) | Reference | 2.69 (1.84, 3.93) | 3.88 (2.88, 5.23) | 8.06 (5.69, 11.41) | <0.0001 |  |
| Old age | 9.96 (5.43, 18.27) | Reference | 1.76 (1.05, 2.94) | 2.82 (1.74, 4.56) | 5.32 (3.24, 8.73) | <0.0001 |  |
| Race |  |  |  |  |  |  | 0.2214 |
| Mexican American | 20.96 (10.49, 41.87) | Reference | 3.45 (1.59, 7.50) | 5.47(2.76,10.84) | 11.84(5.63,24.91) | <0.0001 |  |
| Other Hispanic | 6.63 (3.40, 12.92) | Reference | 2.56 (1.45, 4.54) | 2.13 (1.32, 3.43) | 5.00 (2.91, 8.57) | <0.0001 |  |
| Non-Hispanic White | 11.23 (7.25, 17.38) | Reference | 1.90 (1.35, 2.68) | 3.39 (2.53, 4.54) | 5.83 (4.13, 8.23) | <0.0001 |  |
| Non-Hispanic Black | 15.37 (8.30, 28.45) | Reference | 2.02 (1.37, 2.95) | 3.94 (2.65, 5.88) | 6.69 (4.18, 10.69) | <0.0001 |  |
| Other Race | 19.49 (9.65, 39.36) | Reference | 2.70 (1.31, 5.58) | 3.71 (1.99, 6.91) | 9.74 (5.21, 18.20) | <0.0001 |  |
| Education levels, n(%) |  |  |  |  |  |  | 0.5716 |
| <high school | 13.03 (6.73, 25.20) | Reference | 1.68 (1.01, 2.80) | 3.16 (1.80, 5.56) | 6.12 (3.68, 10.18) | <0.0001 |  |
| =high school | 8.31 (4.84, 14.26) | Reference | 2.13 (1.26, 3.60) | 3.28 (2.10, 5.14) | 5.00 (3.23, 7.73) | <0.0001 |  |
| >high school | 14.26 (8.98, 22.64) | Reference | 2.20 (1.57, 3.08) | 3.61 (2.56, 5.08) | 7.19 (4.99, 10.38) | <0.0001 |  |
| Smoking, n (%) |  |  |  |  |  |  | 0.0130 |
| Current smoker | 7.54 (3.81, 14.93) | Reference | 2.82 (1.29, 6.18) | 4.22 (2.08, 8.54) | 5.66 (2.78, 11.54) | <0.0001 |  |
| Former smoker | 8.27 (4.65, 14.71) | Reference | 1.40 (0.90, 2.18) | 2.31 (1.63, 3.28) | 4.36 (2.81, 6.75) | <0.0001 |  |
| Non-smoker | 17.98 (11.74, 27.55) | Reference | 2.38 (1.83, 3.09) | 4.00 (3.00, 5.33) | 8.56 (6.12, 11.96) | <0.0001 |  |
| Alcohol consumption |  |  |  |  |  |  | 0.6598 |
| No | 11.58 (7.67, 17.49) | Reference | 2.06 (1.44, 2.96) | 3.72 (2.71, 5.10) | 5.89 (4.32, 8.04) | <0.0001 |  |
| Yes | 12.74 (8.50, 19.10) | Reference | 2.12 (1.57, 2.86) | 3.37 (2.50, 4.54) | 6.69 (4.87, 9.20) | <0.0001 |  |
| V/MPA |  |  |  |  |  |  | 0.4247 |
| No | 11.27 (8.03, 15.83) | Reference | 2.04 (1.46, 2.86) | 3.07 (2.35, 4.01) | 6.10 (4.61, 8.07) | <0.0001 |  |
| Yes | 14.09 (8.22, 24.14) | Reference | 2.19 (1.50, 3.20) | 4.23 (2.85, 6.27) | 7.01 (4.57, 10.76) | <0.0001 |  |
| General obesity |  |  |  |  |  |  | 0.6880 |
| Normal | 10.34 (5.15, 20.78) | Reference | 2.44 (1.43, 4.16) | 3.95 (2.30, 6.76) | 5.37 (2.93, 9.84) | <0.0001 |  |
| Overweight | 12.45 (7.79, 19.89) | Reference | 1.95 (1.32, 2.90) | 3.04 (2.18, 4.22) | 6.41 (4.44, 9.25) | <0.0001 |  |
| Obesity | 12.34 (7.29, 20.87) | Reference | 2.05 (1.44, 2.92) | 3.60 (2.59, 5.00) | 6.36 (4.39, 9.22) | <0.0001 |  |
| Abdominal obesity |  |  |  |  |  |  | 0.6468 |
| No | 12.45 (7.85, 19.74) | Reference | 2.28 (1.47, 3.53) | 4.02 (2.68, 6.04) | 6.58 (4.40, 9.82) | <0.0001 |  |
| Yes | 13.88 (9.18, 20.99) | Reference | 2.03 (1.49, 2.76) | 3.34 (2.49, 4.49) | 6.93 (5.04, 9.52) | <0.0001 |  |
| Hypertension |  |  |  |  |  |  | 0.3590 |
| No | 12.90 (8.41, 19.79) | Reference | 1.82 (1.30, 2.53) | 3.10 (2.34, 4.12) | 6.33 (4.54, 8.85) | <0.0001 |  |
| Yes | 11.24 (7.18, 17.59) | Reference | 2.59 (1.72, 3.92) | 4.08 (2.78, 6.00) | 6.64 (4.59, 9.60) | <0.0001 |  |


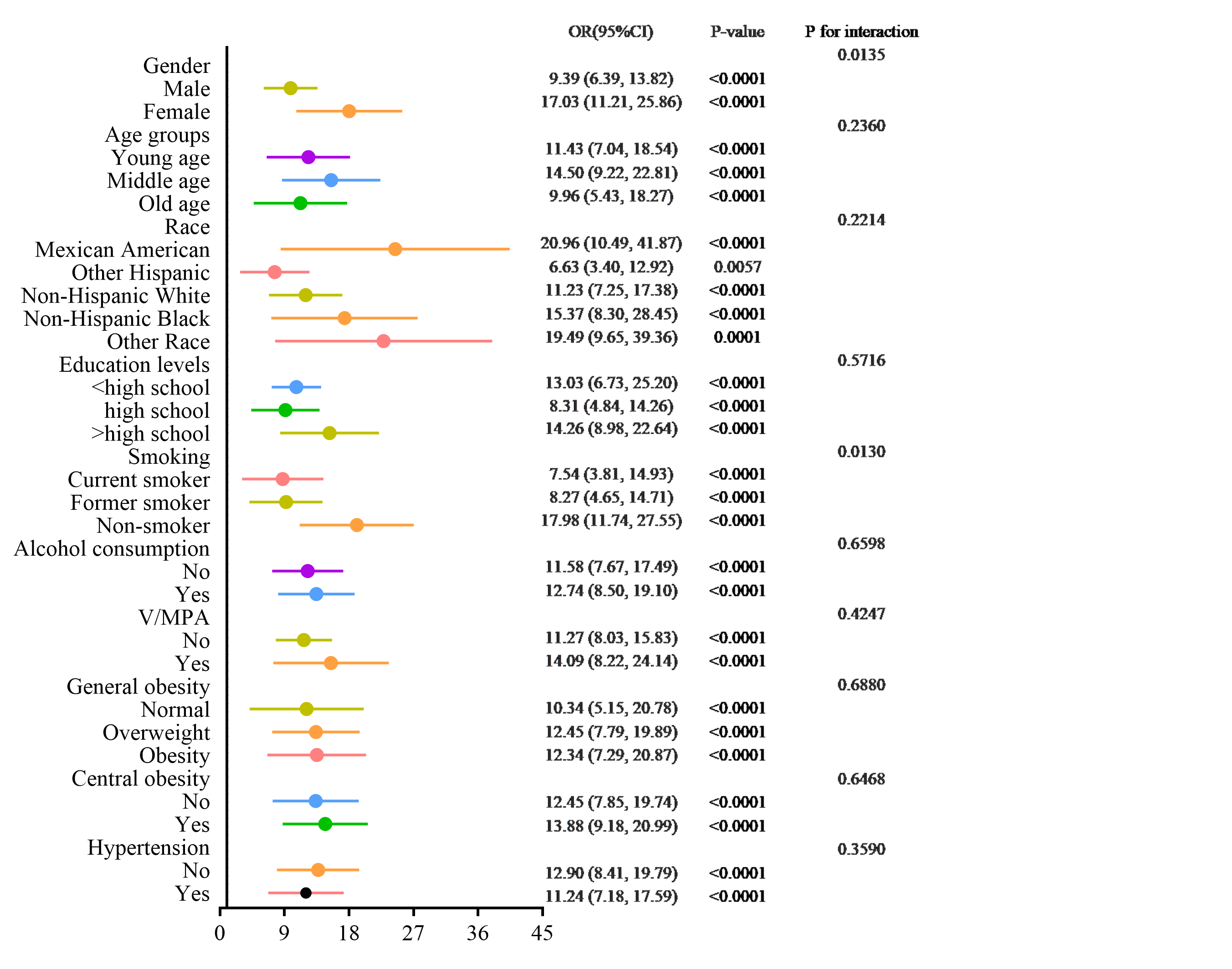


**Fig. S2** Subgroup analysis of AIP and IR


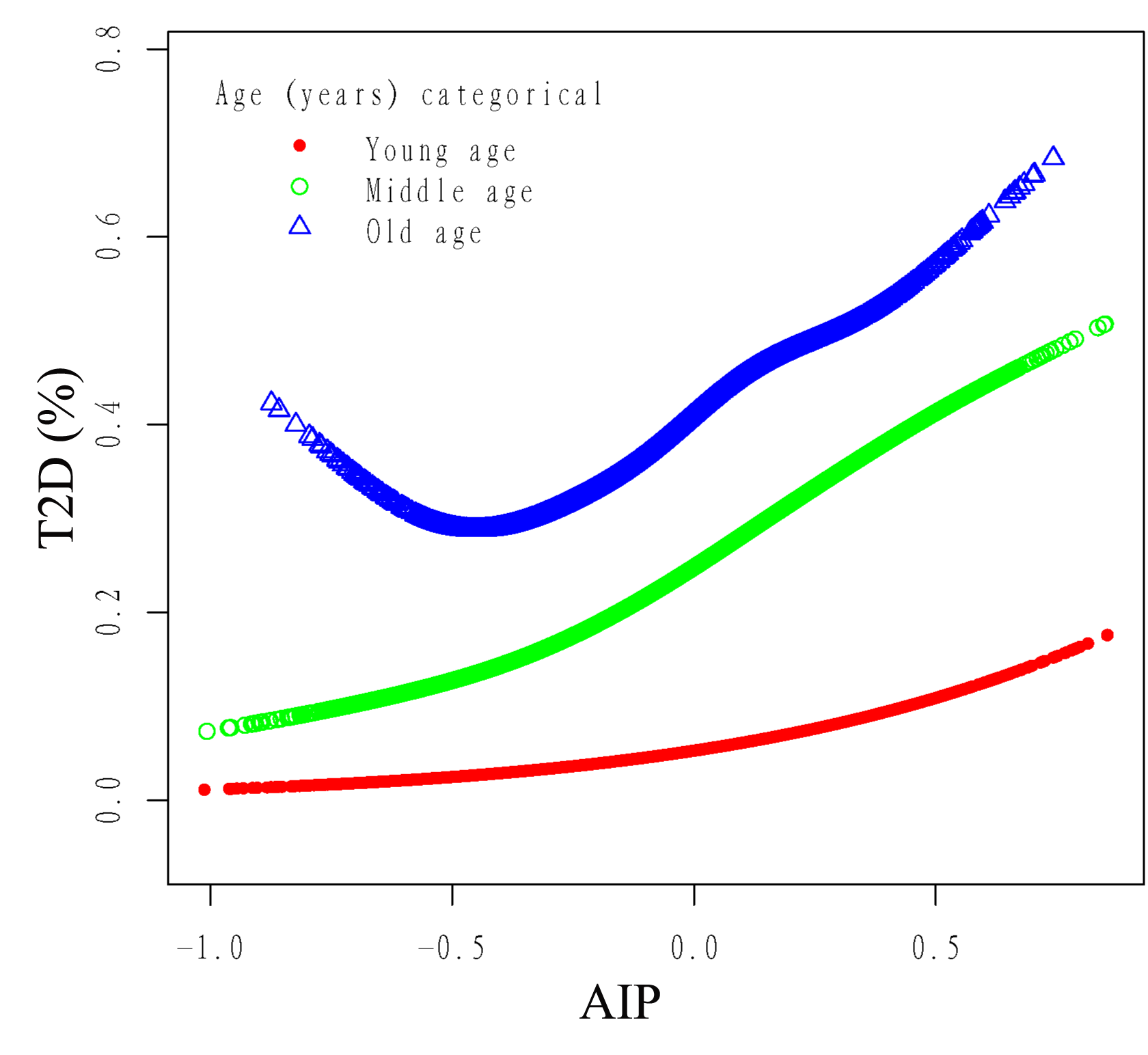

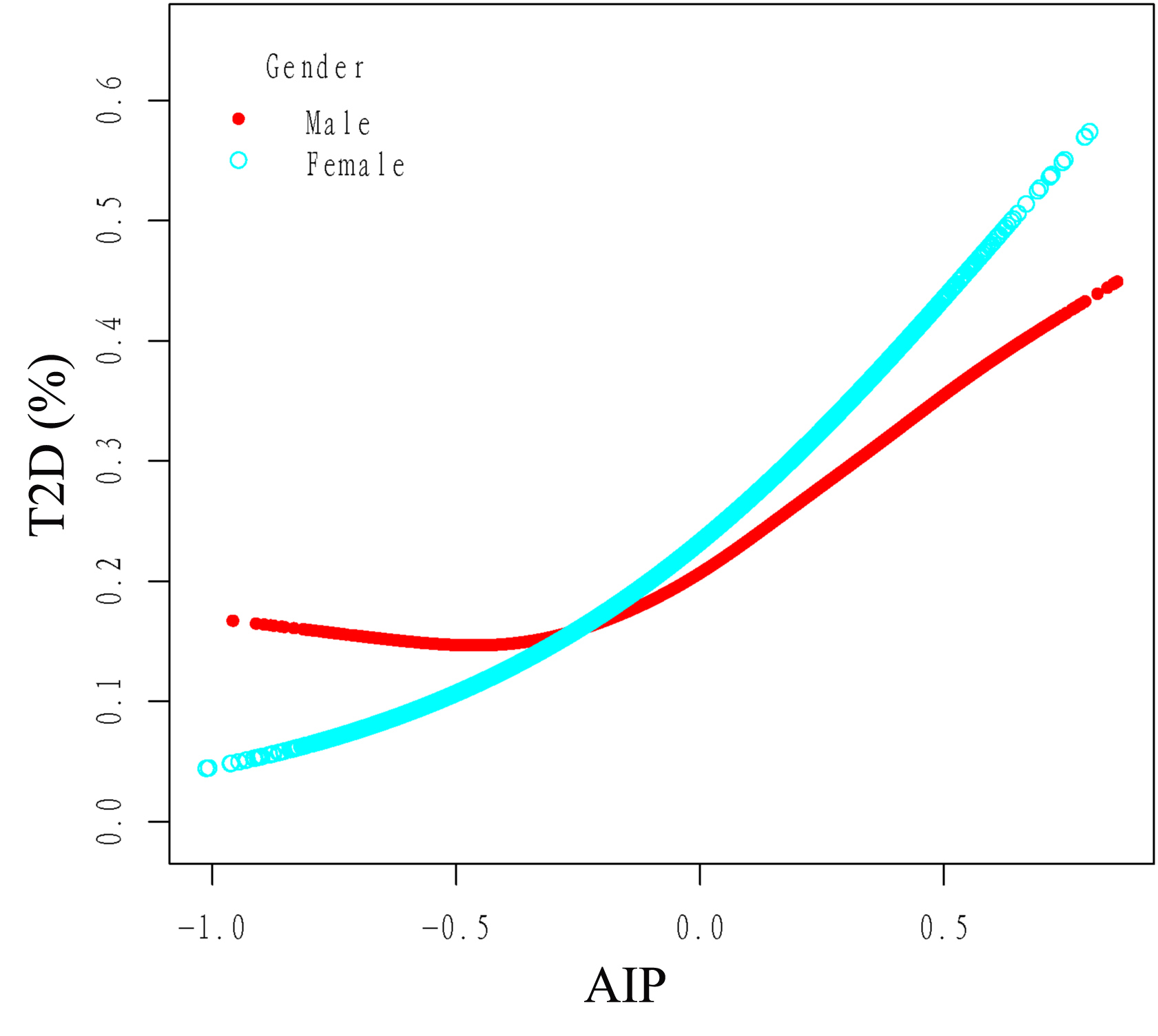


A B


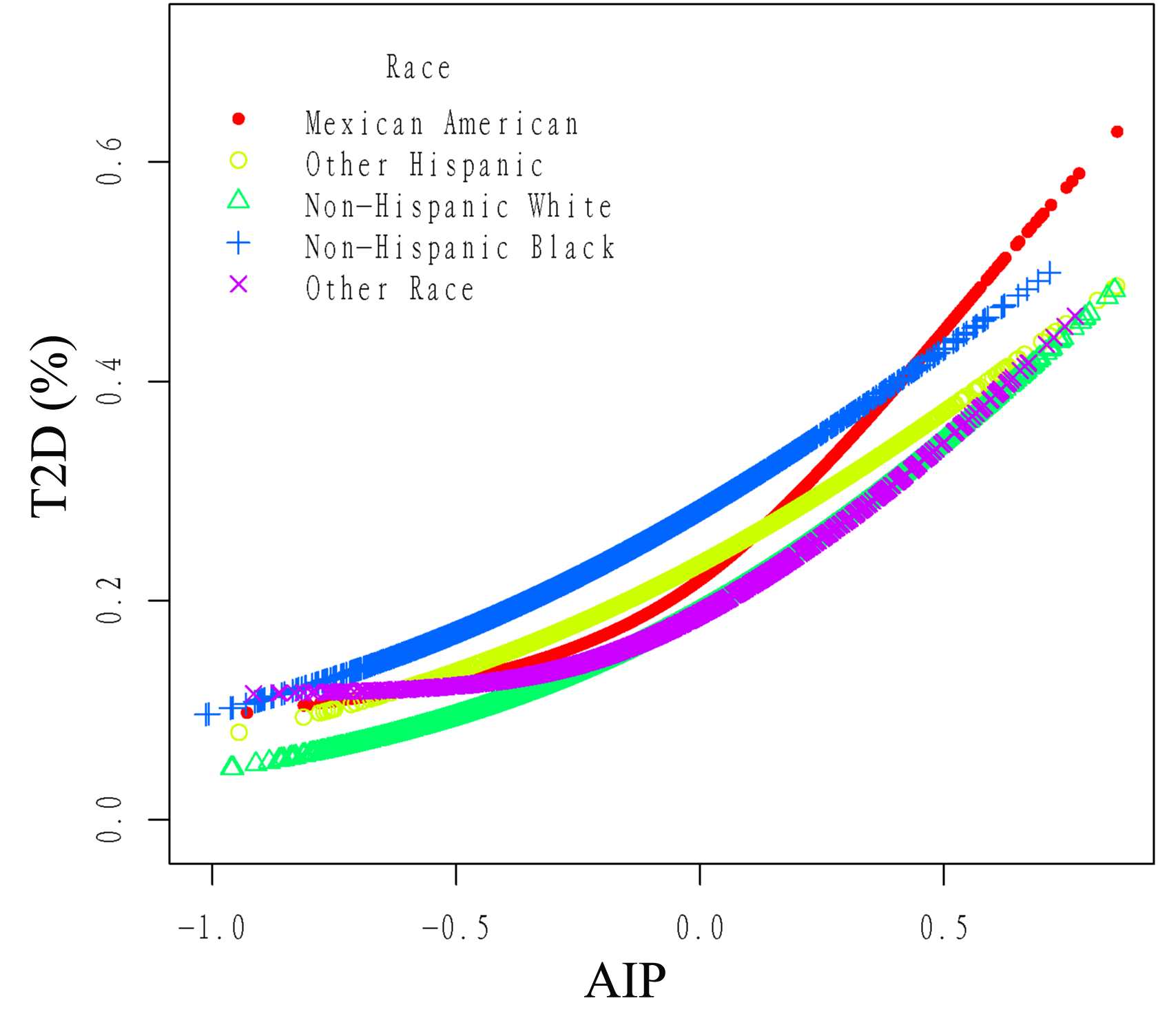

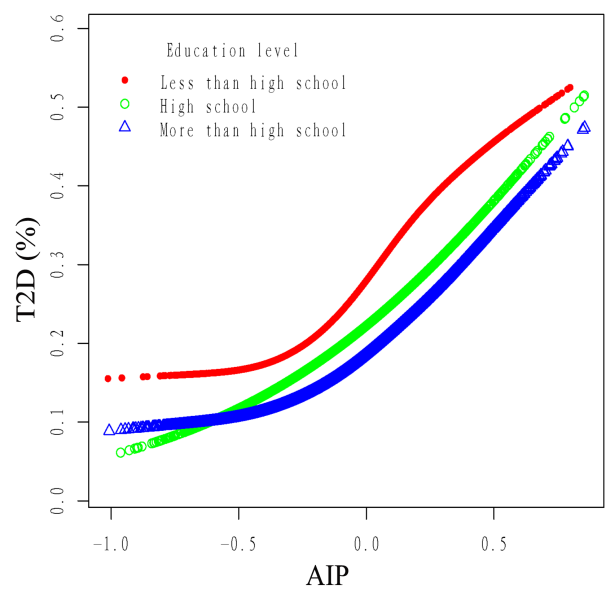


C D


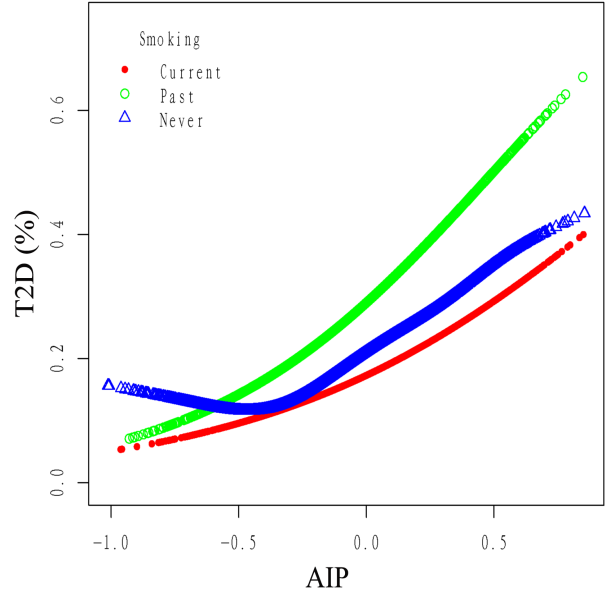

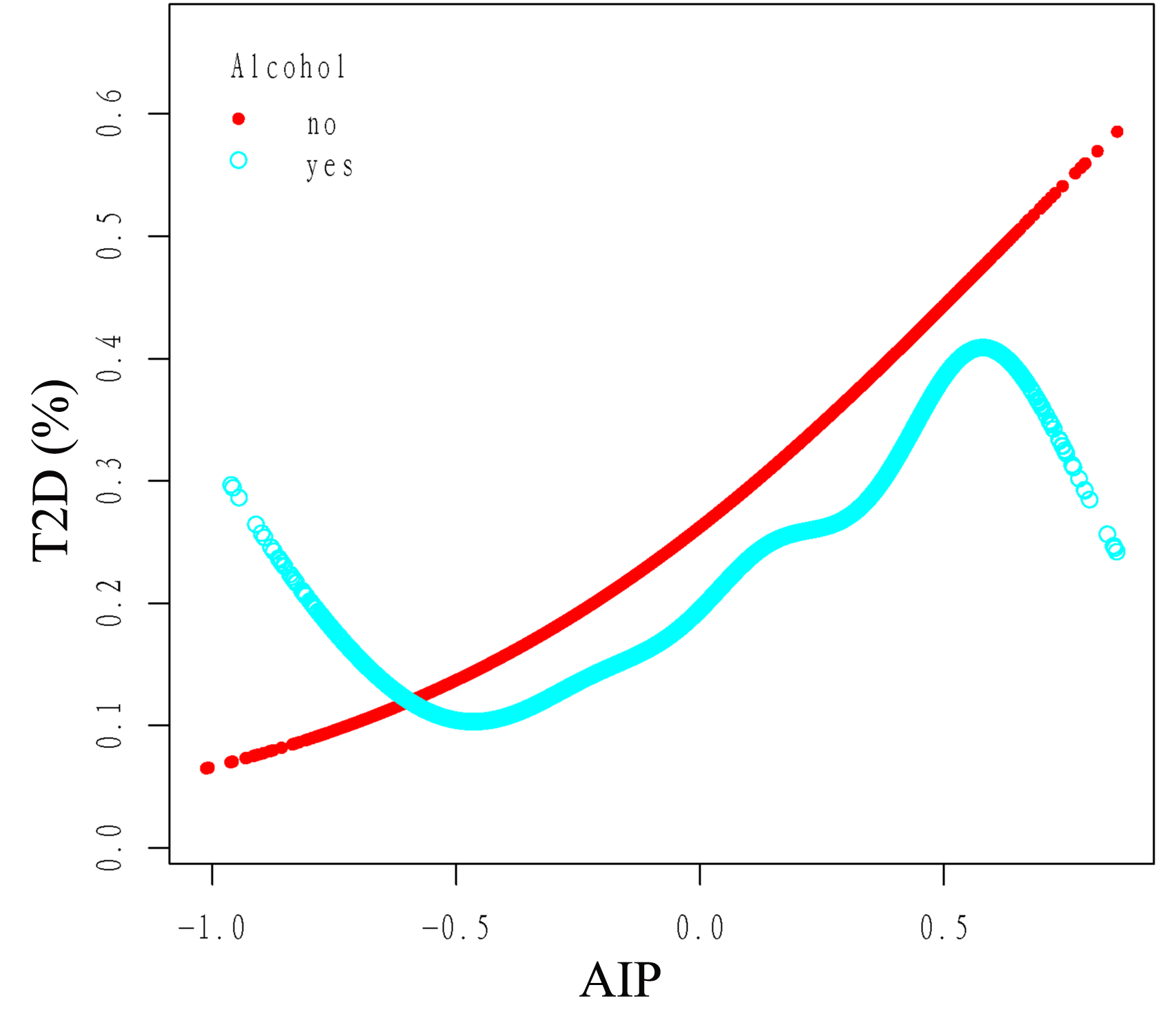


E F


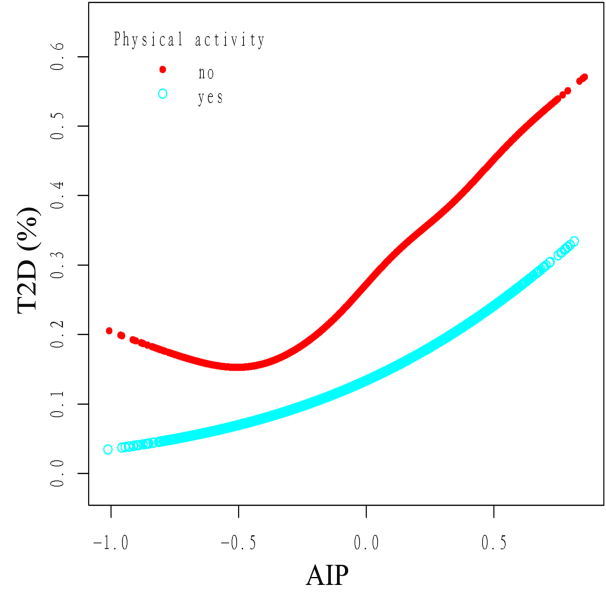

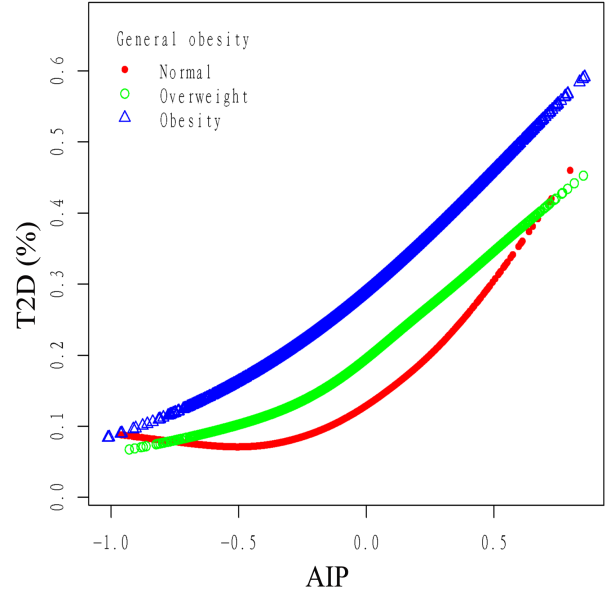


G H


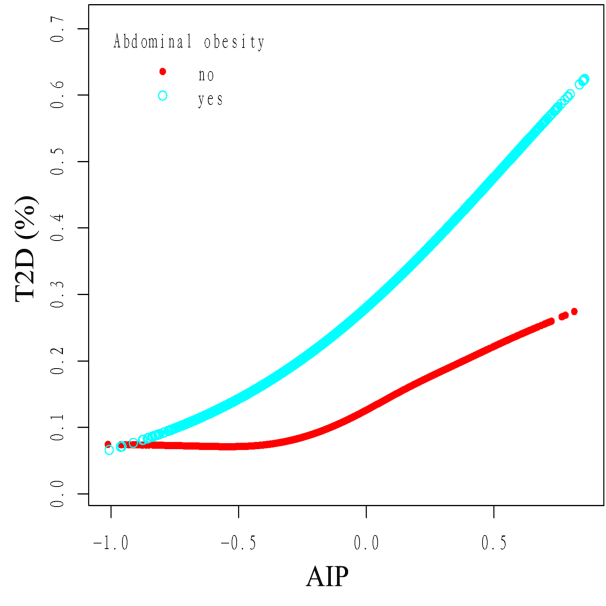

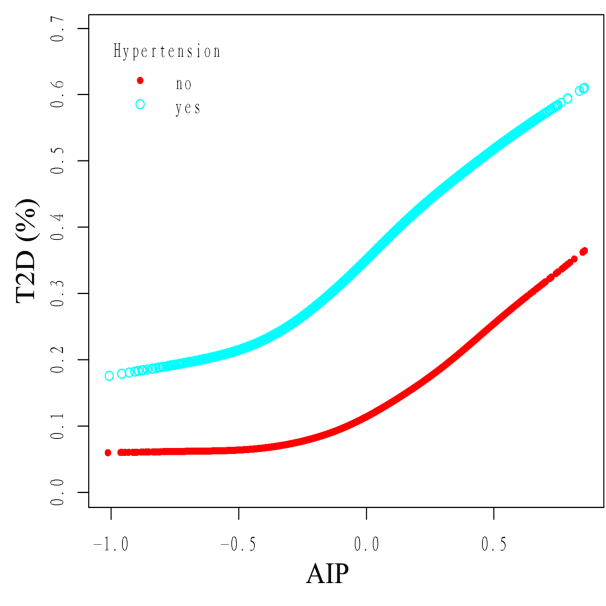


I J

**Fig. S3 Association between AIP and T2D.**

A: Stratified by age; B: Stratified by gender; C: Stratified by race; D: Stratified by education level; E: Stratified by smoking status; F: Stratified by alcohol consumption; G: Stratified by V/MPA; H: stratification by general obesity; I: stratification by central obesity; J: stratification by hypertension.

Adjustment factors: Adjusted for age, gender, race, education level, SBP, DBP, BMI, WC, smoking, alcohol consumption, V/MPA, TC, ALT, γ-GGT, Cr, UA, and Hb. The model was not individually adjusted for age, gender, race, education level, smoking status, alcohol consumption, V/MPA, BMI, WC, SBP, and DBP.

Each line represents a smooth curve fit between variables.


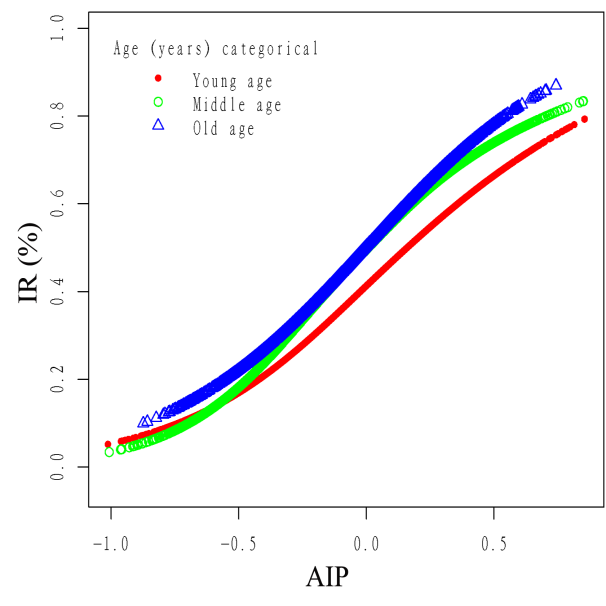

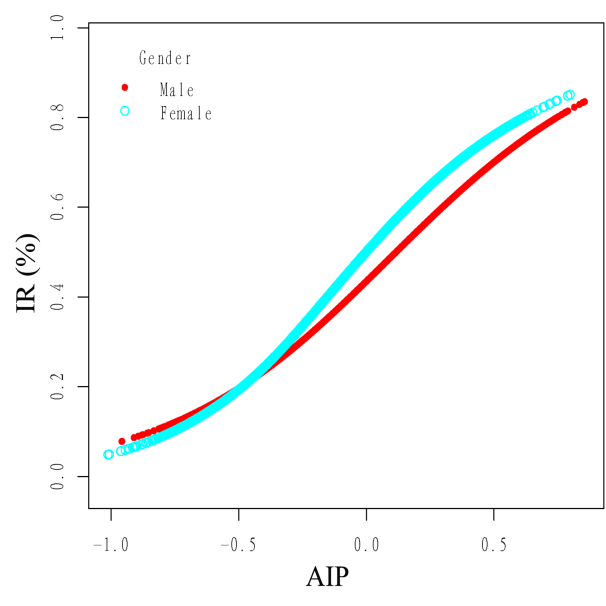


A B


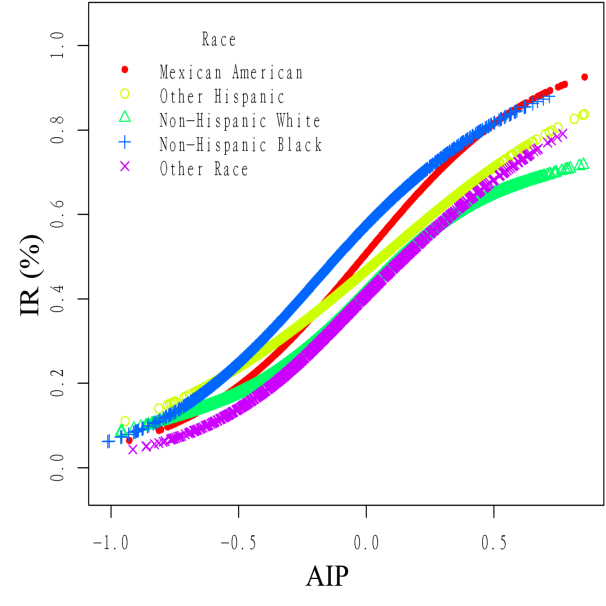

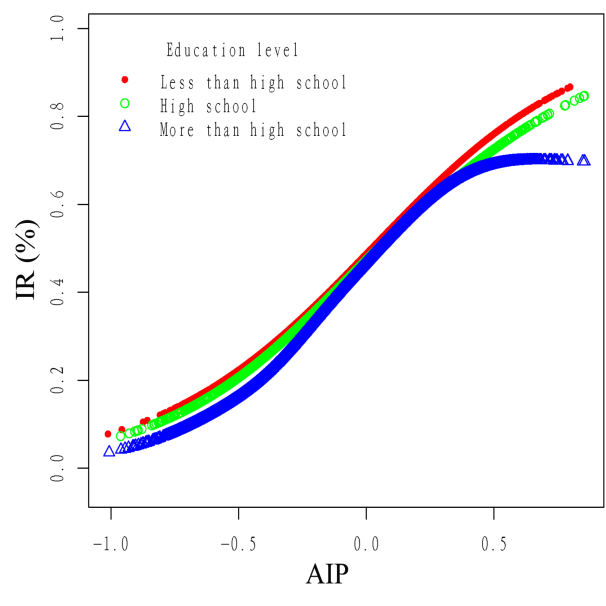


C D


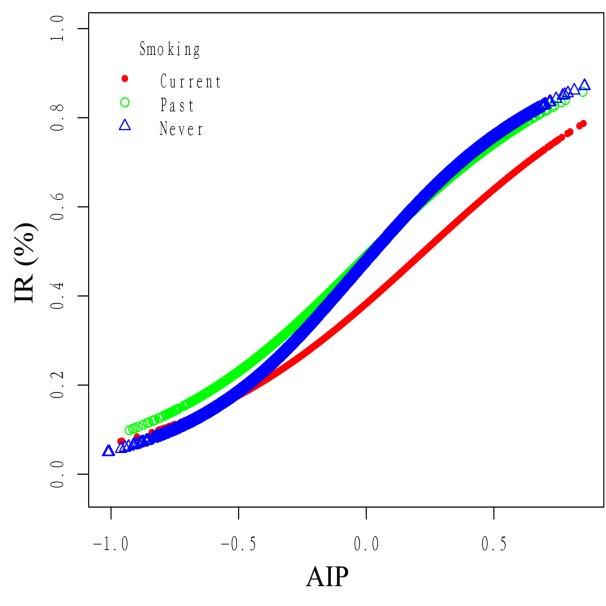

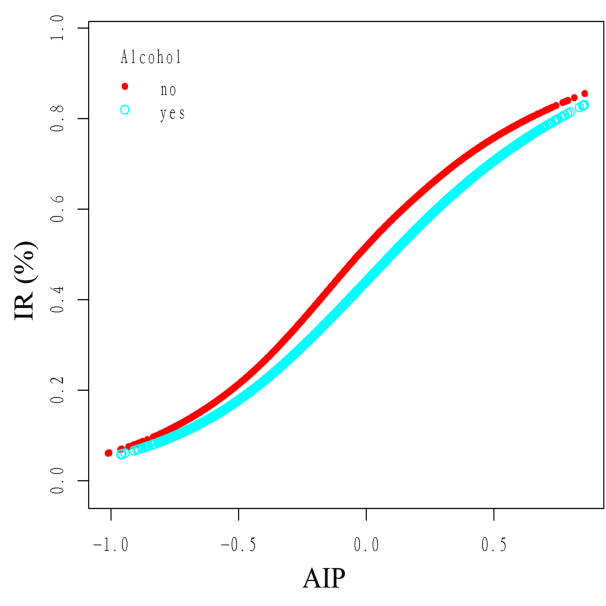


E F


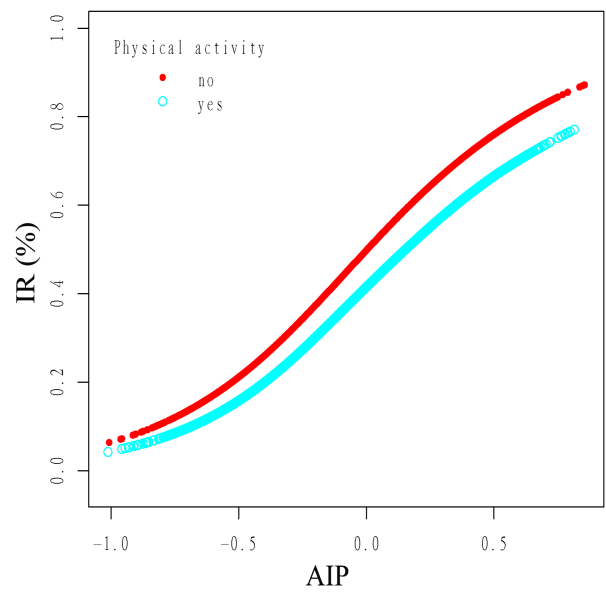

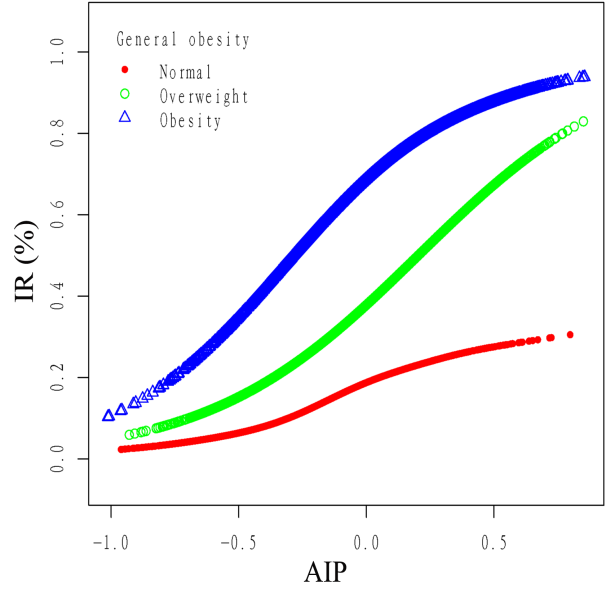


G H


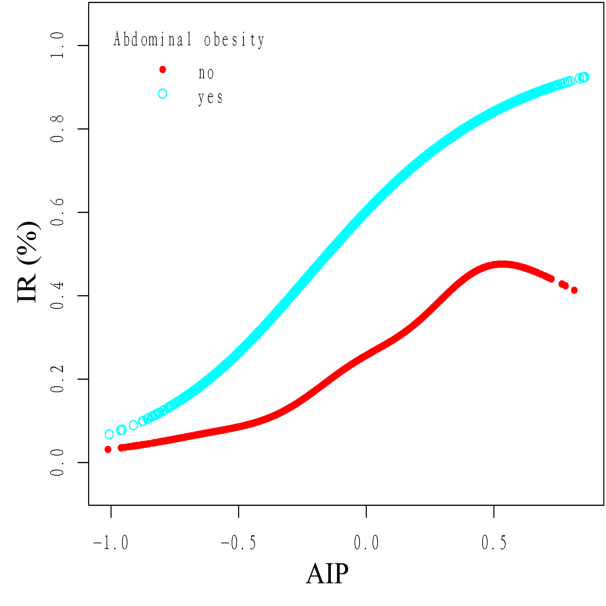

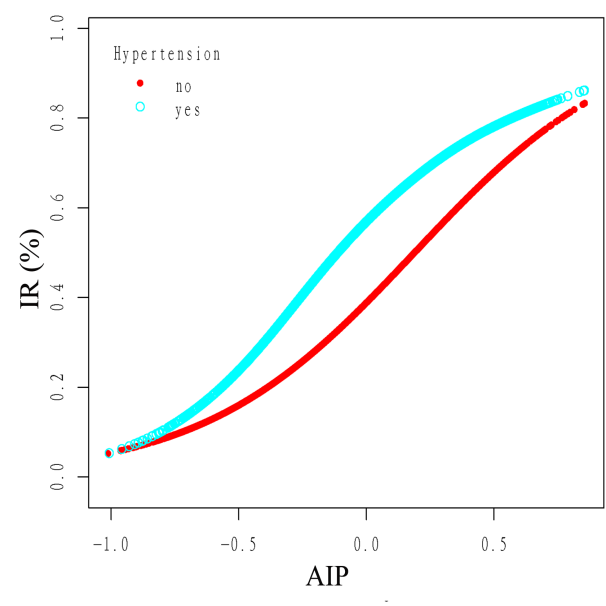


I J

**Fig. S4 Association between AIP and IR.**

A: Stratified by age; B: Stratified by sex; C: Stratified by race; D: Stratified by education level; E: Stratified by smoking; F: Stratified by alcohol consumption; G: Stratified by V/MPA; H: stratification by general obesity; I: stratification by central obesity; J: stratification by hypertension.

Adjustment factors: Adjusted for age, gender, race, education level, SBP, DBP, BMI, WC, smoking, alcohol consumption, vigorous/moderate physical activity, TC, ALT, γ-GGT, Cr, UA, and Hb. The model was not individually adjusted for age, sex, race, education level, smoking, alcohol consumption, vigorous/moderate physical exertion, BMI, WC, SBP, and DBP.

Each line represents a smooth curve fit between variables.
